# DNA methylation mediated by RdDM pathway and demethylation affects furanone accumulation through regulation of *QUINONE OXIDOREDUCTASE* in strawberry

**DOI:** 10.1093/hr/uhad131

**Published:** 2023-07-11

**Authors:** Yunduan Li, Yanna Shi, Yichen Li, Jiao Lu, Yunfan Sun, Yuanyuan Zhang, Wenbo Chen, Xiaofang Yang, Donald Grierson, Zhaobo Lang, Guihua Jiang, Kunsong Chen

**Affiliations:** College of Agriculture & Biotechnology, Zhejiang University, Zijingang Campus, Hangzhou 310058, China; College of Agriculture & Biotechnology, Zhejiang University, Zijingang Campus, Hangzhou 310058, China; Zhejiang Provincial Key Laboratory of Horticultural Plant Integrative Biology, Zhejiang University, Zijingang Campus, Hangzhou 310058, China; The State Agriculture Ministry Laboratory of Horticultural Plant Growth, Development and Quality Improvement, Zhejiang University, Zijingang Campus, Hangzhou 310058, China; College of Agriculture & Biotechnology, Zhejiang University, Zijingang Campus, Hangzhou 310058, China; College of Agriculture & Biotechnology, Zhejiang University, Zijingang Campus, Hangzhou 310058, China; College of Agriculture & Biotechnology, Zhejiang University, Zijingang Campus, Hangzhou 310058, China; College of Agriculture & Biotechnology, Zhejiang University, Zijingang Campus, Hangzhou 310058, China; College of Agriculture & Biotechnology, Zhejiang University, Zijingang Campus, Hangzhou 310058, China; Zhejiang Provincial Key Laboratory of Horticultural Plant Integrative Biology, Zhejiang University, Zijingang Campus, Hangzhou 310058, China; The State Agriculture Ministry Laboratory of Horticultural Plant Growth, Development and Quality Improvement, Zhejiang University, Zijingang Campus, Hangzhou 310058, China; Institute of Horticulture, Zhejiang Academy of Agricultural Sciences, Hangzhou 310021, Zhejiang, China; College of Agriculture & Biotechnology, Zhejiang University, Zijingang Campus, Hangzhou 310058, China; Division of Plant and Crop Sciences, School of Biosciences, University of Nottingham, Sutton Bonington Campus, Loughborough LE12 5RD, United Kingdom; Institute of Advanced Biotechnology and School of Life Sciences, Southern University of Science and Technology, Shenzhen 518055, China; Institute of Horticulture, Zhejiang Academy of Agricultural Sciences, Hangzhou 310021, Zhejiang, China; College of Agriculture & Biotechnology, Zhejiang University, Zijingang Campus, Hangzhou 310058, China; Zhejiang Provincial Key Laboratory of Horticultural Plant Integrative Biology, Zhejiang University, Zijingang Campus, Hangzhou 310058, China; The State Agriculture Ministry Laboratory of Horticultural Plant Growth, Development and Quality Improvement, Zhejiang University, Zijingang Campus, Hangzhou 310058, China

## Abstract

Recently, increasing evidence suggests that DNA methylation plays a crucial role in fruit ripening. However, the role of DNA methylation in regulating specific traits, such as flavor, remains unclear. Here, we report a role of DNA methylation in affecting furanone biosynthesis in strawberry. Strawberry quinone oxidoreductase (FaQR) is a key enzyme in furanone biosynthesis. There are four *FaQR* homologs in strawberry cultivar ‘Yuexin’, and one of them, *FaQR3*, contributes ~50% of *FaQR* transcripts, indicating a major role of *FaQR3* in furanone biosynthesis. Through characterization of levels of DNA methylation and *FaQR3* transcript and furanone contents during fruit ripening and after the application of DNA methylation inhibitor, we found that the DNA methylation level of the *FaQR3* promoter was negatively correlated with *FaQR3* expression and furanone accumulation, suggesting that DNA methylation may be involved in furanone biosynthesis through adjusting *FaQR3* expression, and responded to different temperatures consistently. In addition, transient expression of a gene in the RNA-directed DNA methylation (RdDM) pathway, *FaAGO4*, and enrichment analysis of the 24-nucleotide siRNAs suggested that DNA methylation in the *FaQR3* promoter is mediated by the RdDM pathway. Transient RNA interference (RNAi) of *FaDML* indicated that the demethylation pathway may be involved in regulating furanone accumulation. These findings provide new insights into the role of DNA methylation and demethylation in affecting flavor quality in strawberry during fruit ripening.

## Introduction

Volatile compounds make a remarkably important contribution to fruit flavor and overall quality and directly affect the economic value and, strongly related to consumer preference, overall satisfaction and value [1]. Although volatiles only account for 0.001–0.01% of fruit weight, slight changes can markedly influence fruit taste [1]. Cultivated strawberry (*Fragaria × ananassa*) is a globally popular and economically important horticultural fruit crop with high nutritional value for human health. One of the most attractive features of strawberry fruit is the unique flavor, with more than 360 different volatiles determined in ripe fruit [2]. Volatile compounds in strawberry include esters, alcohols, terpenes, aldehydes, furans, lactones, organic acids, and aromatic hydrocarbons [3]. Of these, furanone is one of the characteristic aroma compounds distinguishing strawberry from other fruits and is significantly positively correlated with the overall flavor intensity [4]. Furanone is described as a strong, sweet, caramel-like, and fruity/floral aroma, including 4-hydroxy-2,5-dimethyl-3(2)H-furanone (HDMF) and 2,5-dimethyl-4-methoxy-3(2)H-furanone (DMMF). In fruits of tomato (*Solanum lycopersicum*), grape (*Vitis vinifera*), and raspberry (*Rubus idaeus*), furanone is found only in a trace amount [5]. Furanone is particularly abundant in pineapple and strawberry [5], and strawberry is a vital model for non-climacteric fruits and has been used successfully for exploring the regulation of furanone biosynthesis. Furthermore, understanding the factors controlling furanone biosynthesis is of great significance for improving flavor quality in strawberry.

So far, substantial progress has been made in the elucidation of the biological pathway leading to furanone [5]. d-Fructose-1,6-diphosphate is a natural precursor of furanone [6]. 4-Hydroxy-5-methyl-2-methylene-3(2H)-furanone (HMMF) is the immediate precursor of HDMF. HMMF is catalyzed by strawberry quinone oxidoreductase (FaQR) to produce the aroma-active compound HDMF [7]. Subsequently, HDMF is catalyzed by *O*-methyltransferase (FaOMT) to form DMMF [8], and also can be metabolized to the flavorless 2,5-dimethyl-4-hydroxy-2H-furan-3-one glucoside (HDMF-glucoside) by the UDP-dependent glycosyltransferase (UGT71K3) [9]. Ultimately, HDMF-glucoside is further catalyzed to HDMF malonyl-glucoside at late stages of fruit ripening. As the key enzyme that limits furanone biosynthesis, FaQR is mainly accumulated at the late stages of strawberry development and ripening, paralleling furanone accumulation in the fruit [7]. Previous studies have reported that a FaERF#9-FaMYB98 transcription complex activates the *FaQR* promoter to regulate furanone biosynthesis in strawberry [10]. With the rapid development of headspace solid-phase microextraction (HS-SPME) technology and the publication of the octoploid strawberry genome [11], more and more studies of genetic mechanisms of volatile compound synthesis have been performed in cultivated strawberries [1]. However, the regulatory mechanism of furanone biosynthesis has not been well understood.

DNA methylation exists in all cytosine sequence contexts: CG, CHG, and CHH (H = A, T, or C) in plants. In *Arabidopsis thaliana*, DNA methylation is regulated by four processes, including *de novo* DNA methylation mediated by the RNA-directed DNA methylation pathway (RdDM), DNA methylation maintenance, active DNA demethylation mediated by ROS1, and passive DNA demethylation [12]. Cytosines in three sequence contexts can be *de novo* methylated by the RdDM pathway, in which 24-nucleotide (nt) siRNAs guide the DNA methyltransferase DRM2 to methylate specific target loci [13]. At some activated transposons, POL II and RDR6 collaboratively produce precursors of 21/22-nt siRNAs that mediate DNA methylation similarly to 24-nt siRNAs [12]. DNA methylation can be maintained during replication, and cytosine methylation maintenance depends on different cytosine sequence contexts. CG and CHG cytosine methylation are maintained through the action of MET1 (Methyltransferase 1) and CMT3 (Chromomethylase 3), respectively, whereas, the maintenance of CHH methylation depends on CMT2 (Chromomethylase 2) and the RdDM pathway [12]. In plants, active DNA demethylation is initiated by the ROS1 (Repressor of Silencing 1) family of bifunctional 5-methylcytosine DNA glycosylases-apurinic/apyrimidinic lyases through a base excision repair pathway [14].

As a common epigenetic modification in plants and animals, DNA methylation is involved in multiple aspects of plant growth and development, including gene imprinting, seed development, fruit development and ripening, biotic and abiotic stresses, vegetative growth and pattern formation, etc. [12]. Recently, increasing reports indicate that DNA methylation modification plays a vital role in fruit development and ripening in horticultural crops. Currently, the tendency towards variation of genomic DNA methylation has been characterized during fruit development and ripening in several fruits [15], such as tomato [16], strawberry [17], and sweet orange [18], although for horticultural crops most studies on DNA methylation have been carried out in tomato. A naturally occurring epigenetic mutation with hypermethylation in the *CNR* promoter inhibits tomato fruit ripening [19]. In tomato, the fruit ripening process is accompanied by global DNA hypomethylation due to increased expression of *SlDML2*, a close homolog of ROS1, and silencing of *SlDML2* inhibits fruit ripening [16, 20, 21]. In postharvest storage, low-temperature stress repressed expression of *SlDML2* and increased the DNA methylation of promoters of fruit flavor-related genes, leading to a loss of flavor in tomato [22]. These studies revealed an important role of DNA methylation modification during fruit development and ripening. However, the genetic basis of the influence of DNA methylation on specific traits is less understood.

Up to now, the function of DNA methylation in the formation of flavor quality in strawberry has not been reported. In our study, the effect of DNA methylation in *FaQR*-catalyzed furanone biosynthesis was investigated. Among the four *FaQR* homologs from octoploid strawberry ‘Yuexin’, the promoter of *FaQR3* showed a reduction in DNA methylation during the strawberry fruit ripening process, which is negatively correlated with the transcript level of *FaQR3* and furanone content. In addition, similar results were obtained in fruits treated with DNA methylation inhibitor versus mock-treated fruit and fruit stored at 20 versus 10°C. Weakened RdDM activity is reported to be associated with overall decline of genomic DNA methylation during the strawberry fruit ripening process. Consistently, transient expressing or silencing of *FaAGO4* and 24-nt siRNA analysis suggested that the RdDM pathway is crucial for regulating DNA methylation in the *FaQR3* promoter. Additionally, transient RNA interference (RNAi) of *FaDML1*/*2* implied that demethylation is also involved in DNA methylation modification of the *FaQR3* promoter.

## Results

### Expression levels of four *FaQR* homologs during fruit ripening in octoploid strawberry

Four *FaQR* homologs, denominated *FaQR1*, *FaQR2*, *FaQR3*, and *FaQR4* (called *FaQR* previously [10]), were characterized in ‘Yuexin’ cultivated strawberry using genome walking. These four *FaQR* homologs were confirmed to be present in different collections of octoploid strawberries using PCR amplifications (Supplementary Data Table S1). The corresponding promoters (*FaQR1Pro*, *FaQR2Pro*, *FaQR3Pro*, and *FaQR4Pro*) of these four homologs shared a 253-bp sequence in common upstream of the initiation codon (ATG), but the remaining sequences were very different (Supplementary Data Fig. S1). Genome analysis of octoploid strawberry revealed that *Fragaria vesca* and *F. iinumae* are two of the diploid progenitor species of cultivated octoploid strawberry (*F.* × *ananassa*) [23]. Through BLASTing the published *F. vesca* and *F. iinumae* strawberry genomes, we found that *FaQR3* originated from the subgenome of *F. vesca*, and *FaQR4* came from *F. iinumae*. The source of the remaining two homologs remains unknown. By analyzing the corresponding gene coding sequences of these four homologs, we found that the coding sequence driven by *FaQR3Pro* was most similar to *FvQR* (Supplementary Data Fig. S2), and the protein sequence of *FaQR3* was consistent with that in *F. vesca* (Supplementary Data Fig. S3). The other three genes encode an identical protein.

**Figure 1:**
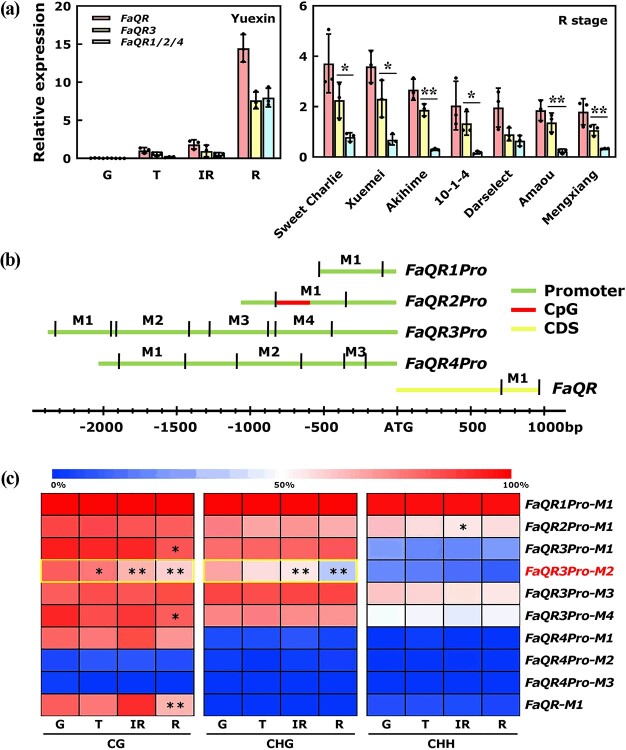
DNA methylation levels of four *FaQR* promoters during fruit ripening in ‘Yuexin’ strawberry. **a** Relative expression levels of *FaQR3* and *FaQR1/2/4* during fruit ripening in ‘Yuexin’ strawberry and R stages of seven cultivated octoploid strawberries. G, T, IR, and R refer to green, turning, intermediate red, and full red stage, respectively. Bars refer to standard deviation from three biological repetitions. ^*^.01 < *P* < .05, ^**^P < .01; one-way ANOVA. **b** Detected region in four *FaQR* promoters. M1–M4 represent the detected region in the promoter where CG sites are highly concentrated. **c** DNA methylation levels of four *FaQR* promoters from whole ‘Yuexin’ fruit. Data used to generate the heat map were from three biological repetitions. A total of 36 independent clones for each fragment were sequenced. The heat map was made using the absolute percentage of methylation derived from bisulfite sequencing. When analyzing significant differences, the DNA methylation pattern of the promoter at each stage was compared with that in G stage.

**Figure 2:**
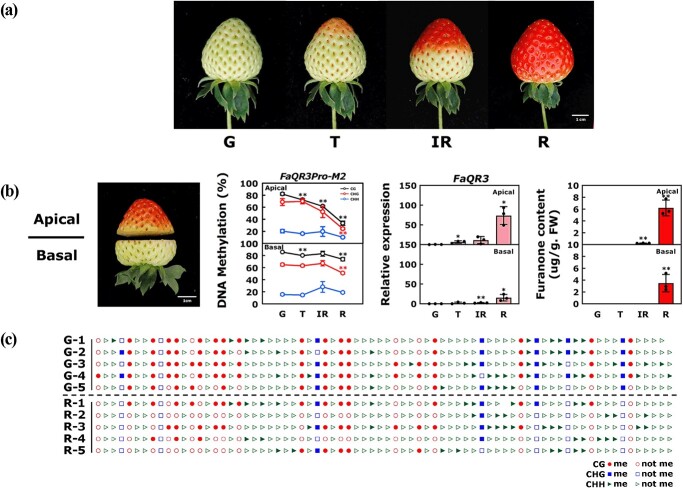
Changes in DNA methylation in *FaQR3Pro-M2*, *FaQR3* expression, and furanone content in the apical and basal section during ‘Yuexin’ strawberry ripening. **a** ‘Yuexin’ fruits at four ripening stages. **b** Changes in *FaQR3Pro-M2* DNA methylation, *FaQR3* expression, and furanone content. Bars stand for standard deviation from three biological repetitions. FW, fresh weight. ^*^Significant differences using one-way ANOVA analysis. Significant differences in DNA methylation in the promoter, *FaQR3* expression, and furanone content at each stage are compared with those at G stage. **c** Changes in DNA methylation sites in *FaQR3Pro-M2* region at G and R stages. Schematic diagram shows representative sequencing results for five single colonies in the apical section from bisulfite sequencing.

The coding sequences driven by different promoters shared high similarity, with only a few single-nucleotide polymorphisms (SNPs) in the 5′ UTR (Supplementary Data Fig. S4). Based on the SNPs, we could distinguish FaQR3Pro-driven transcripts from the remaining transcripts (Supplementary Data Fig. S4). We analyzed the four transcripts driven by FaQR3Pro and FaQR1/2/4Pro during different stages of the fruit development and ripening process in ‘Yuexin’ strawberry and R (full red) stages of seven other cultivated octoploid strawberries, including ‘Sweet Charlie’, ‘Xuemei’, ‘Akihime’, ’10-1-4’, ‘Darselect’, ‘Amaou’, and ‘Mengxiang’. Overall, ~50% of the total transcripts of *FaQR*s were from *FaQR3*, indicating that *FaQR3* contributes the most transcripts of *FaQR*s (Fig. 1a). Additionally, specific primers were designed in the same region with identical sequences of four *FaQR* homologs to detect the proportion of *FaQR*s. The proportion of four *FaQR* homologs at each stage was analyzed by sequencing single colonies (Supplementary Data Fig. S4d). It was found that the *FaQR3* sequence accounts for ~50% of the total sequences, which supported the results of RT–qPCR.

### Changes of DNA methylation in *FaQR3Pro* during strawberry fruit ripening

Bisulfite sequencing was used to test the DNA methylation patterns within promoters of the four *FaQR* genes (Fig. 1b) at four fruit development and ripening stages [green (G), turning (T), intermediate red (IR), and full red (R)] in ‘Yuexin’ strawberry fruits. The DNA methylation levels of *FaQR1Pro*, *FaQR2Pro*, and *FaQR3Pro* were higher than that of *FaQR4Pro* in the detected regions (Fig. 1c), using whole fruit samples. Among the detected regions of the four promoters, the M2 section (a region between −1910 and −1418 bp upstream of ATG) of the *FaQR3* promoter (*FaQR3Pro-M2*) showed a significant reduction of mCG and mCHG during fruit ripening (Fig. 1c). From the G stage to the R stage, mCG in *FaQR3Pro-M2* decreased from ~82.31 to ~59.39%, mCHG decreased from ~66.43 to ~37.06%, and mCHH decreased from ~20.33 to ~10.02% (Fig. 1c). However, other detected regions of *FaQR* promoters showed limited changes of the DNA methylation patterns. The downregulation trend of DNA methylation in the *FaQR3Pro-M2* region is consistent with previously published whole-genome bisulfite sequencing in strawberry [17] (*F. × ananassa* Duch. cv. ‘Hongjia’) (Supplementary Data Fig. S5). Strawberry fruits start to ripen in the apical section (Fig. 2a). We divided strawberry fruits into two parts, an apical section and a basal section (calyx end), and checked the DNA methylation level of *FaQR3Pro-M2*, *FaQR3* gene expression, and furanone content at four ripening stages. During the fruit ripening process, the tendency to variation in DNA methylation in *FaQR3Pro-M2* was more obvious in the apical sections than in the basal sections. mCG and mCHG in the *FaQR3Pro-M2* of the fruit apical section decreased from ~81.81 and ~ 68.56% at the G stage to ~33.5 and ~ 24.28% at the R stage, respectively. However, in the later-ripening basal sections, mCG and mCHG only decreased to ~73.74 and ~ 50.79%, respectively, at the R stage. Consistent with the change in DNA methylation, *FaQR3* transcript levels and furanone accumulation were highly upregulated in apical sections (Fig. 2b). Interestingly, also in the apical sections, the levels of mCG and mCHG showed a rapid reduction from IR to R, which is consistent with a rapid accumulation of *FaQR3* expression and furanone at the ripening stage. Additionally, furanone content exhibited a rapid rise from 0.246 μg/g at the IR stage to 6.204 μg/g at the R stage in the fruit apical section (Fig. 2b), indicating that DNA methylation is coordinated with furanone accumulation during strawberry fruit ripening. *FaQR3Pro-M2* is a region between −1910 and −1418 bp upstream of ATG, and many DNA methylation sites in this region were demethylated from the G to the R stage in the fruit apical section (Fig. 2c).

### DNA methylation of *FaQR3Pro-M2* is associated with *FaQR3* expression and furanone content

Since *FaQR3* originated from the *F. vesca* subgenome, the DNA methylation level of *FvQRPro-M2* was measured at four *F. vesca* fruit ripening stages (Supplementary Data Fig. S6). mCG and mCHG in *FvQRPro3-M2* showed a decreasing trend in *F. vesca*, while *FvQR* expression and furanone showed an increasing trend, suggesting this pattern of DNA methylation has been conserved during the evolution of octoploid strawberry. Besides ‘Yuexin’ strawberry fruits, we observed similar changes in DNA methylation, *FaQR3* expression, and furanone accumulation patterns (Supplementary Data Fig. S6) in other octoploid cultivated strawberry cultivars (’10-1-4’, ‘Akihime’, ‘Xuemei’, and ‘Sweet Charlie’) during fruit ripening (Supplementary Data Fig. S7), suggesting that the association between DNA methylation and furanone synthesis exists in different strawberry cultivars.

**Figure 3:**
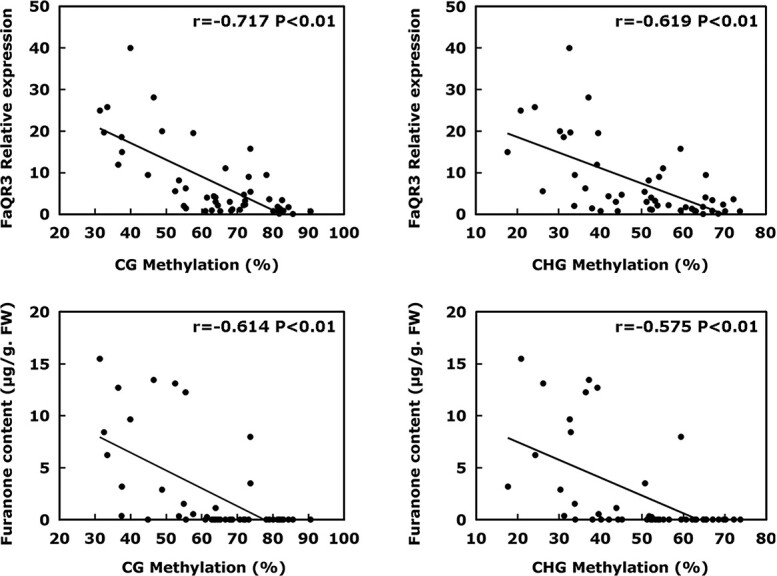
Linear regression analysis of methylation level of *FaQR3Pro-M2* with *FaQR3* expression and furanone content. Analysis of significant differences was carried out via SPSS Statistics 22.0. FW, fresh weight.

Moreover, DNA methylation of *FaQR3Pro-M2* was negatively correlated with *FaQR3* gene expression during fruit ripening in these strawberry cultivars (mCG correlation: *r* = −.717, *P* < .01; mCHG correlation: *r* = −.619, *P* < .01) (Fig. 3). *FaQR3Pro-M2* DNA methylation was also negatively correlated with furanone content (mCG correlation: *r* = −.614, *P* < .01; mCHG correlation: *r* = −.575, *P* < .01).

### Changes in DNA methylation of *FaQR3Pro-M2* during fruit ripening at different storage temperatures

**Figure 4:**
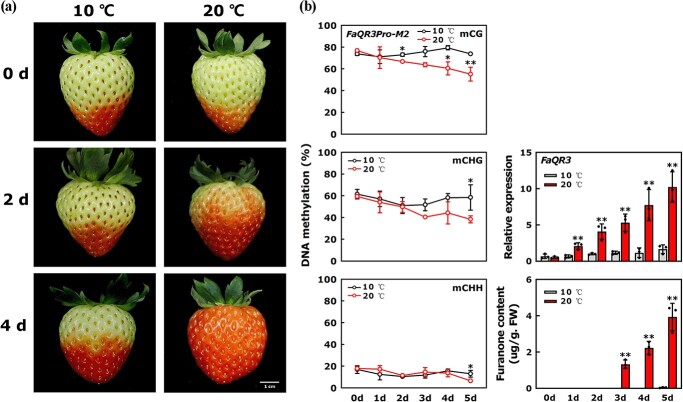
Treating postharvest ‘Yuexin’ strawberry fruits at 10 and 20°C. **a** Pictures of temperature-treated strawberries. **b** Methylation level of *FaQR3Pro-M2*, *FaQR3* expression, and furanone content after 10 and 20°C treatment. Bars stand for standard deviation from three biological repetitions. FW, fresh weight. When analyzing the significant differences, the data from postharvest storage at 20°C were compared with those at 10°C. Asterisks indicate significant differences using one-way ANOVA.

Low-temperature treatment is widely used in postharvest storage. DNA methylation usually responds to environmental stimuli, including temperature, light, drought, high salinity, and so on [12]. To explore the effect of temperature on the DNA methylation pattern of *FaQR3Pro-M2*, we treated postharvest ‘Yuexin’ strawberry fruits at T stage under 10 and 20°C, respectively (Fig. 4a). Under 10°C treatment, the ripening process was slowed, and strawberries at 20°C gradually ripened with the extension of the time and reached the R stage on the 4th day (Fig. 4a). At 10°C, the DNA methylation of *FaQR3Pro-M2* and *FaQR3* expression remained basically unchanged, and furanone content could hardly be detected in fruits treated on the 5th day, which is consistent with the slowed fruit ripening process (Fig. 4b). In contrast, at 20°C, mCG and mCHG of *FaQR3Pro-M2* were reduced, and *FaQR3* expression and furanone accumulation increased greatly with increasing treatment time at 20°C (Fig. 4b). On the whole, treatment at 20°C could effectively promote demethylation of *FaQR3Pro-M2*, followed by upregulation of *FaQR3* expression and furanone, indicating that DNA methylation-mediated furanone biosynthesis is sensitive to different storage temperatures.

### Treatment with 5-azacytidine promotes fruit ripening and furanone accumulation in strawberry.

**Figure 5:**
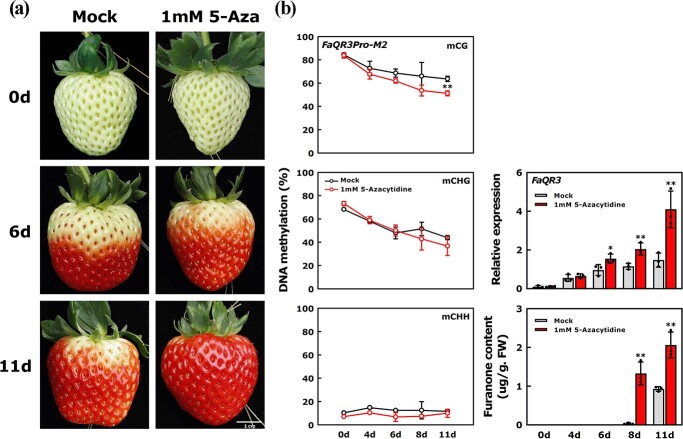
Treating ‘Yuexin’ strawberry fruits with DNA methylation inhibitor. **a** Treatment with DNA methylation inhibitor. Fruits treated with ddH_2_O served as control (mock). 5-Aza, 5-azacytidine. **b** Changes in *FaQR3Pro-M2* methylation, *FaQR3* expression, and furanone content after treatment. Bars stand for standard deviation from three biological repetitions. FW, fresh weight. When analyzing the significant differences, the data from the 1 mM 5-azacytidine treatment were compared with the control. Asterisks indicate significant differences using one-way ANOVA.

To further investigate the significance of DNA methylation for furanone synthesis during strawberry ripening, we treated ‘Yuexin’ strawberry fruits with 1 mM 5-azacytidine (Fig. 5), and sampled fruits on the 0th, 4th, 6th, 8th and 11th days, respectively. Compared with mock treatment, more abundant color accumulation was observed in treated fruits on the 6th day after treatment (Fig. 5a). mCG of *FaQR3Pro-M2* began to decrease slightly on the 4th day in treated fruits, and decreased to ~51.18% on the 11th day (Fig. 5b). Compared with mock controls, the upregulation of *FaQR3* expression and furanone accumulation were enhanced by the 5-azacytidine treatment. On the whole, 1 mM 5-azacytidine treatment promoted fruit ripening, which was preceded by a decrease in mCG and mCHG level in the *FaQR3Pro-M2* region of the promoter, higher *FaQR3* expression and an increase in furanone accumulation, implying that the alteration of DNA methylation in *FaQR3Pro-M2* may affect *FaQR3* transcription to alter furanone biosynthesis.

### Transient expression analysis of *FaAGO4*, *FaDML1*, and *FaDML2*

**Figure 6:**
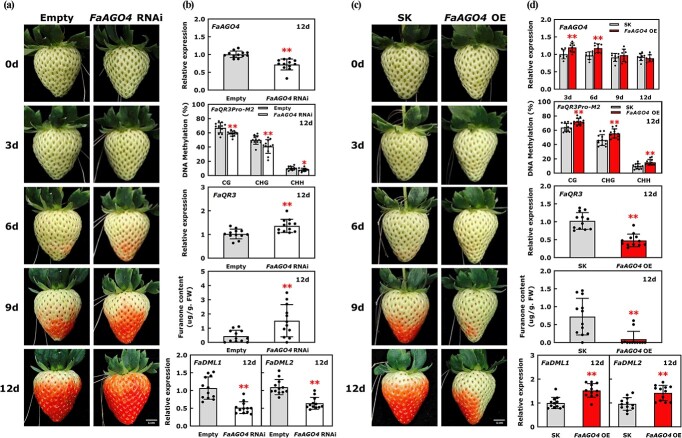
Transient RNAi and overexpression of *FaAGO4* affect furanone biosynthesis. **a** Transient RNAi of *FaAGO4*. ‘Empty’ represents empty pHellsgate vector. **b** RT–qPCR assays of *FaAGO4*, *FaQR3*, *FaDML1*, and *FaDML2*, methylation level of *FaQR3Pro-M2*, and furanone quantification in *FaAGO4* RNAi fruit and control fruit. **c** Transient overexpression of *FaAGO4*. SK represents empty pGreen II 0029 62-SK vector. **d** RT–qPCR assays of *FaAGO4*, *FaQR3*, *FaDML1*, and *FaDML2*, methylation level of *FaQR3Pro-M2*, and furanone quantification in *FaAGO4* OE fruit and control fruit. Error bars stand for standard deviation based on 12 biological repetitions. Asterisks indicate significant differences using one-way ANOVA analysis. FW, fresh weight.

RdDM-mediated DNA methylation is a vital DNA methylation establishment pathway in plants, and it involves siRNA biogenesis and siRNA-guided DNA methylation. Previous studies have shown that a reduced RdDM pathway contributes to DNA hypomethylation during fruit ripening in strawberry [17]. Small-RNA analysis using published data [17] indicated that 24-nt siRNAs were enriched in *FaQR3Pro-M2* and were reduced during fruit ripening, while few 21/22-nt siRNAs were enriched in *FaQR3Pro-M2* and their content did not change significantly during fruit ripening (Supplementary Data Fig. S8), implying that the change in DNA methylation in *FaQR3Pro-M2* can be mediated by the RdDM pathway involving 24-nt siRNAs. To further investigate the effect of the RdDM pathway on furanone synthesis, we carried out transient RNAi of *FaAGO4* by injection of *Agrobacterium tumefaciens* into strawberry fruits at the G stage. The transcript levels of *FaAGO4* were reduced to ~70% by transient RNAi, indicating that the transient silencing system was effective (Fig. 6b). On the 6th day after treatment, *FaAGO4* RNAi fruit showed a large area of red accumulation, while the control fruits were only slightly tinted (Fig. 6a). In addition, the total content of furanone in *FaAGO4* RNAi fruits was up to 3.49-fold higher compared with the control fruits (Fig. 6b), suggesting that the transient RNAi of *FaAGO4* promoted furanone biosynthesis and fruit ripening in strawberry. On the 12th day after infiltration, mCG, mCHG, and mCHH levels of *FaQR3Pro-M2* were markedly reduced in *FaAGO4* RNAi fruits compared with the control fruits (Fig. 6b). Transient silencing of *FaAGO4* also enhanced *FaQR3* expression (Fig. 6b). These results implied that the RdDM-mediated DNA methylation pathway is involved in furanone accumulation in strawberry.

In contrast, transient overexpression (OE) of *FaAGO4* significantly inhibited fruit ripening (Fig. 6c). On the 3rd day after injection, the expression level of *FaAGO4* increased by ~20% in *FaAGO4* OE fruits relative to control fruits (Fig. 6d). However, at longer times after infiltration the *FaAGO4* expression in OE fruit gradually returned to the normal level. On the 12th day after injection, the control fruits were close to the FR stage, while *FaAGO4* OE fruits were not uniformly colored and had not reached the IR stage. Compared with the control fruits, the mCG, mCHG, and mCHH levels of *FaQR3Pro-M2* remained high and *FaQR3* expression was significantly suppressed in *FaAGO4* OE fruits (Fig. 6d). The total furanone content in *FaAGO4* OE fruits was only ~13% of that in control fruit, supporting a critical role for the RdDM pathway in furanone accumulation.

**Figure 7:**
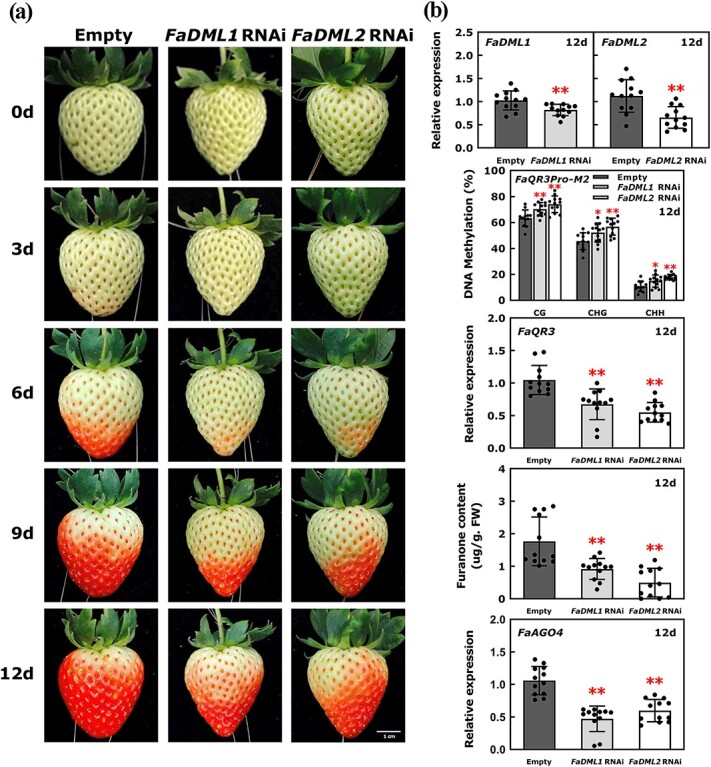
Transient silencing of *FaDML1* and *FaDML2* affects furanone biosynthesis. **a** Transient RNAi of *FaDML1* and *FaDML2*. Empty represents empty pHellsgate vector. **b** RT–qPCR assays of *FaDML1*, *FaDML2*, *FaQR3*, and *FaAGO4*, methylation level of *FaQR3Pro-M2*, and furanone quantification in *FaDML1* or *FaDML2* RNAi fruit and control fruit. Error bars stand for standard deviation based on 12 biological repetitions. Asterisks indicate significant differences using one-way ANOVA analysis. FW, fresh weight.

In plants, the RdDM-mediated DNA methylation pathway and ROS1-mediated DNA demethylation pathway can oppose each other to regulate DNA methylation patterns in the genome [14]*.* Phylogenetic analysis of the ROS1 family proteins from strawberry (*F. × ananassa*), *S. lycopersicum*, and *A. thaliana* indicated that *FaDML1* and *FaDML2* were close to *AtROS1* (Supplementary Data Fig. S9). We explored the potential functions of these two *FaDML* genes in strawberry fruit ripening and furanone synthesis by carrying out transient RNAi for *FaDML1* and *FaDML2,* respectively (Fig. 7). The transient silencing of either *FaDML1* or *FaDML2* inhibited strawberry fruit ripening (Fig. 7a) and the transcript levels of *FaDML1* and *FaDML2* were reduced to ~80 and ~ 58% of those in control fruits, respectively (Fig. 7b). On the 6th day after infiltration, the *FaDML1* and *FaDML2* RNAi fruits just began to change color, while the control fruits were approaching the IR stage (Fig. 7a). On the 12th day after injection, the methylation levels of *FaQR3Pro-M2* in *FaDML1* and *FaDML2* RNAi fruits remained high, and *FaQR3* transcript levels were reduced to ~64 and ~ 53% of those in control fruits, respectively. In *FaDML1* and *FaDML2* RNAi fruits, the furanone content was reduced to ~52 and ~28% of that in control fruits, respectively, suggesting that the DNA demethylation pathway is also important for furanone biosynthesis. Interestingly, *FaDML1* and *FaDML2* expression was increased in *FaAGO4* OE fruit (Fig. 6d); likewise, *FaDML1* and *FaDML2* expression was markedly reduced in *FaAGO4* RNAi fruits (Fig. 6b). In addition, we found that *FaAGO4* expression was reduced to ~45 and ~ 56% in *FaDML1* and *FaDML2* RNAi fruits (Fig. 7b). These results suggested that the RdDM pathway might interact with the DNA demethylation pathway to co-regulate the DNA methylation pattern and furanone biosynthesis in strawberry.

## Discussion

### FaQR3 is the main quinone oxidoreductase regulating furanone biosynthesis during strawberry fruit ripening

The cultivated commercial strawberry (*F. × ananassa*), an allo-octoploid (2*n* = 8*x* = 56), emerged from the hybridization of two wild octoploid progenitors ~300 years ago [11]. The two progenitors, *Fragaria chiloensis* and *Fragaria virginiana* were formed by the merger of four diploid progenitor species [24]. Until now, there has been some controversy about the origin of cultivated strawberries [25]. Diploid *F. iinumae* and *F. vesca* have been commonly identified as the progenitors of the octoploid cultivated strawberry *F. × ananassa* [26], but whether additional diploid progenitors contributed to the octoploid strawberry genome remains under debate [23]. In this study, we obtained four *FaQR* homologs from ‘Yuexin’ cultivated strawberry. While BLASTing to the newly published octoploid strawberry genome [11] and several published diploid genomes [23], we identified the origin of two *FaQR* homologs. *FaQR3* came from the subgenome of *F. vesca* and *FaQR4* came from *F. iinumae*. The source of the remaining two genes is still unknown. The coding sequence driven by *FaQR3Pro* was most similar to that of *FvQR* (Supplementary Data Fig. S2), and the protein sequence of *FaQR3* was consistent with that in *F. vesca* (Supplementary Data Fig. S3). The coding sequences driven by the remaining three promoters shared high similarity and encoded proteins with identical amino acid sequences. This result was similar to that obtained by the reanalysis of genomes using a chromosome-scale phylogenomic approach [25]. *Fragaria iinumae* was the closest diploid progenitor of the remaining two subgenomes, and an unsampled population or extinct populations of *F. iinumae* comprised the progenitors of cultivated strawberry. This provides a reasonable explanation for the fact that three other genes encode the same amino acid sequence. After ancient allopolyploid events, a dominant subgenome contributed by the *F. vesca* progenitor was identified [11]. The single dominant subgenome contained markedly greater biased exchanges between homoeologous chromosomes, gene content, and gene expression abundance, compared with the other subgenomes. Certain metabolic pathways, including those involved in strawberry color, aroma, and flavor, were largely controlled by the dominant subgenome, *F. vesca* [11]. This view is consistent with our conclusion that the expression of *FaQR3* originating from *F. vesca* accounted for ~50% of the total transcripts of *FaQR*s during fruit ripening, indicating it is vital for the regulation of furanone biosynthesis. Meanwhile, transcripts of *FaQR1/2/4* were induced during fruit ripening, indicating they also contribute to furanone accumulation. In this study, the DNA methylation patterns of the four *FaQR* promoters were different within the observed regions during strawberry fruit ripening. On the whole, the DNA methylation of *FaQR1Pro* and *FaQR2Pro* within the detected regions maintained a relatively high level. DNA methylation in *FaQR4Pro* within the detected regions was very low except for mCG of *FaQR4Pro-M1*. mCG and mCHG in *FaQR3Pro-M2* were substantially downregulated during fruit ripening. A previous study has reported that an FaERF#9-FaMYB98 transcription complex activated the *FaQR4* promoter, and thus regulated furanone biosynthesis in strawberry [10]. Taken together, these results suggested that transcriptional and epigenetically regulation are involved in determining the transcripts of *FaQR*s. Additionally, mCG and mCHG of *FaQR3Pro-M2* also decreased during fruit ripening in *F. vesca*, consistent with many octoploid cultivated strawberries (Supplementary Data Fig. S6), indicating this modification mechanism of DNA methylation in *FaQR3Pro* may originate from diploid progenitor *F. vesca*.

### DNA methylation in *FaQR3Pro-M2* contributes to transcriptional control of *FaQR3*

Until now, there has been increasing evidence that DNA methylation modification is vital for fruit development and ripening process in horticultural crops. In addition to plant hormones and transcription factors (TFs), DNA methylation is also vital for fruit development and ripening, and may interact with hormone-related and other TFs [27, 28]. Much evidence indicates that DNA methylation is related to the regulation of fruit quality in horticultural crops, but only the effect of DNA methylation on color is relatively clear. It is well known that DNA methylation at the *MYB10* promoter has been confirmed as a regulator of anthocyanin biosynthesis. This epigenetic modification mechanism has been extensively studied in many horticultural crops, including apple [29], orange [30], pear [31], and peach [32]. In this study, the importance of reduced methylation in *FaQR3Pro-M2* for furanone biosynthesis during fruit ripening was clarified using a variety of different approaches, including the use of four fruit ripening stages, apical and basal sections of fruits, different cultivars, DNA methylation inhibitor treatment, and transient expression of FaAGO4. Besides, *cis*-acting elements were enriched in the *FaQR3Pro-M2* region that underwent loss of DNA methylation during ripening (Supplementary Data Fig. S10), suggesting a potential function of this M2 region in *FaQR3* transcription. In these *cis*-acting elements, the DNA methylation levels of ABRE, CGTCA/TGACG-motif and G-box decreased during fruit ripening. A previous study reported that DNA methylation in the promoter can directly repress the gene transcription process via suppressing the binding of transcription activators [12]. We screened 72 fruit-expressed *AP2/ERF*s, 69 fruit-expressed *MYB*s and 17 ripening-related TFs, and measured their abilities to transactivate *FaQR3Pro* (Supplementary Data Fig. S11) by dual-luciferase assays, which showed that 20 TFs would increase the promoter activity of *FaQR* up to 2-fold (Supplementary Data Fig. S10), while the activity of the truncated promoter (lacking the M2 region) of *FaQR3* displayed lower transactivation activities by 17 TFs (Supplementary Data Fig. S10), suggesting that the *FaQR3Pro-M2* region is important for *FaQR3* transcription. However, we did not find *cis*-elements for *ERF*, *ARF*, and *RAV* in the M2 region, which also could transactivate the *FaQR3* promoter. TFs can be directly or indirectly recruited to their targets [10], so we speculate that these TFs may regulate the promoter of *FaQR3* via other mediators.

In addition to the programmed changes during development and ripening, postharvest storage also affects fruit quality. In tomato, cold storage leads to a reduction in flavor quality [22] and low-temperature stress results in a major variation in DNA methylation status in the tomato genome. Many methylation changes occur in promoters of genes involved in flavor synthesis, fruit quality, and ripening. DNA methylation in promoters of flavor-associated genes can be affected by low temperature, resulting in reduced flavor-associated volatile content [22]. In our study, the methylation of *FaQR3Pro-M2* in harvested fruits stored at 10°C basically remained unchanged, leading to relatively low expression and an extremely low furanone content. It is possible that this DNA methylation mechanism associated with relatively low gene expression is an additional mechanism against unnecessary gene expression in this low-temperature stress response. Interestingly, the expression of the other three *FaQR* homologs, *FaQR1*, *FaQR2*, and *FaQR4*, was also upregulated at 20°C (Supplementary Data Fig. S12), but their promoter DNA methylation levels within the detected regions were not significantly reduced at 20°C, implying their transcription might not be regulated by DNA methylation modification during postharvest storage at different temperatures. Taking these results together, changes in the methylation status of *FaQR3Pro-M2* have a major effect on strawberry fruit flavor quality by adjusting *FaQR3* transcription during fruit ripening.

### Potential function of RdDM and demethylation pathways in furanone biosynthesis

In *A. thaliana*, the DNA methylation dynamic pattern is regulated by DNA methylation and active demethylation [33] and the dynamic balance between DNA methylation and demethylation can affect the genome-wide DNA methylation level [34]. A 39-bp DNA methylation monitoring sequence (MEMS) within the *ROS1* promoter could sense DNA methylation and demethylation activities and states to coordinate genome-wide DNA methylation by adjusting the expression pattern of *ROS1* [33]. Strawberry undergoes an overall decrease of DNA methylation during the fruit ripening process because of a reduction in RdDM pathway activity [17]. Many ripening-regulated gene promoters exhibit hypomethylation at the mature (ripening) stage, including genes involved in anthocyanin accumulation, production of flavor volatiles, chlorophyll biosynthesis, etc. Our study revealed that mCG and mCHG in *FaQR3Pro-M2* are positively related to the expression of *FaAGO4* and the enrichment of 24-nt siRNA in the region of *FaQR3Pro-M2* during fruit ripening, suggesting the potential impact of RdDM pathways in furanone accumulation. In *A. thaliana*, *ROS1* resists the RdDM pathway in order to prevent genomic DNA hypermethylation. The RdDM pathway regulates the DNA methylation pattern of MEMS within the *ROS1* promoter to control *ROS1* expression, and *ROS1* expression is inhibited in mutants defective in the RdDM pathway, implying the pattern and activities of DNA methylation and demethylation are coordinated [33]. Our study is similar to some results in *A. thaliana*. In strawberry, decreased methylation was caused by transient silencing of *FaAGO4* versus control, which was accompanied by lower *FaDML1/2* expression. Meanwhile, the delayed reduction of methylation in *FaQR3Pro-M2* was caused by transient overexpression of *FaAGO4* compared with control, which was accompanied by induced *FaDML1/2* expression (Fig. 6), suggesting changes in the RdDM pathway can affect demethylation during fruit ripening.

Previous studies have shown that SlDML2-mediated DNA demethylation is vital for tomato fruit ripening [20]. The *sldml2* mutation inhibits fruit ripening with loss of flavor and pigment. Similar to the above results, the *FaDML*-mediated DNA demethylation pathway also plays a vital role during strawberry fruit ripening (Fig. 7). Furthermore, the decreased demethylation caused by transient silencing of *FaDML1* or *FaDML2* results in weakening of the RdDM pathway, suggesting changes in demethylation can affect the RdDM pathway. The RdDM pathway may also antagonize *ROS1* to prevent genome-wide DNA hypermethylation.

The DNA methylation state of MEMS in the *ROS1* promoter is also regulated via ROS1-dependent active demethylation in *A. thaliana*. MEMS shows an increased DNA methylation in *ROS1* mutants, concomitant with enhanced *ROS1* expression [33]. *ROS1* expression relies on hypermethylation in MEMS generated by the RdDM pathway, revealing that DNA methylation and active demethylation are dynamically coordinated. The methylation-sensitive regulatory mechanism of *ROS1* expression has also been identified in other plants, such as rice and maize [12], implying this regulation mechanism may be conserved for regulating the dynamic balance of DNA methylation in plants [12]. Taking these results together, there may also be an adjustable dynamic between RdDM-mediated DNA methylation and the demethylation pathway in strawberry fruit (Supplementary Data Fig. S13).

## Materials and methods

### Plant materials

Nine cultivated types of octoploid strawberry (*F.* × *ananassa*) were used in this study (Supplementary Data Table S1), including ‘Yuexin’, ‘Camarosa’, ‘10-1-4’, ‘Sweet Charlie’, ‘Amaou’, ‘Akihime’, ‘Xuemei’, ‘Mengxiang’, and ‘Darselect’. The leaves of ‘Camarosa’ were provided by Jiangsu Academy of Agricultural Sciences. The remaining cultivated strawberries were planted in the orchard of Zhejiang Academy of Agricultural Sciences. Strawberries were grown in the field in a tunnel greenhouse covered with plastic film. *Fragaria vesca* was grown and preserved in our laboratory. Fruits at four fruit ripening stages were harvested, including G (green), T (turning), IR (intermediate red), and R (full red). Each biological replicate consisted of six fruits. Three biological repetitions were sampled. After removing the calyces, ‘Yuexin’, ‘Akihime’, ‘Xuemei’, ‘Sweet Charlie’, ’10-1-4’ and *F. vesca* were cut into apical and basal sections and sampled separately. Achenes remained on the fruits. All the samples were rapidly divided into small pieces and immediately frozen with liquid nitrogen, and stored at −80°C for further assays.

### Detection of DMMF and HDMF

For the detection of HDMF and DMMF in strawberry fruits at different ripening stages, an automated HS-SPME and a liquid-injection system equipped with sample injector CTC Pal ALS was applied as described previously [35]. One gram of powdered fruit stored at −80°C and 2 ml 20% sodium chloride were homogenized in a 10-ml centrifuge tube. Then 20 μl 0.766 μg/μl 2-octanol and 300 μl of CH_2_Cl_2_ were added to the tube to extract the volatiles. After homogenization, the tube was placed at indoor temperature and subsequently centrifuged for 5 min. The subnatant was transferred into a new 1.5-ml tube containing 20 mg anhydrous Na_2_SO_4_. Finally, 1 μl of sample was injected through the CombiPAL autosampler. HDMF and DMMF were quantified and confirmed by comparing with the retention time of injected standards (Sigma–Aldrich). Based on a total ion chromatogram (TIC), the quantitative analysis of both HDMF and DMMF was performed using the peak area of the internal standard as a reference.

### Gene isolation, promoter cloning, and analysis

*FaQR* promoters were obtained using genome walking as described previously [36]. The *cis*-acting regulatory elements in the 1910-bp *FaQR3* promoter were predicted via PlantCARE. FaMYBs and FaDMLs in strawberry were identified using hidden Markov model (HMM) profiles and Pfam databases [37]. The TFs used in this study were cloned from ‘Yuexin’ by referring to the *Fragaria × vesca* genome. Accession numbers used in this study are shown in Supplementary Data Table S2.

### Construction of phylogenetic tree

Multiple sequence alignments were carried out through DNAMAN. A phylogenetic tree of DML proteins from strawberry (*F. × ananassa*), *S. lycopersicum*, and *A. thaliana* was established in MEGA 6.0 through the neighbor-joining method [37].

### Small RNA analysis

The data obtained from a previously published article were used for 24-nt siRNA analysis [17]. The Trimmomatic tool [38] was used to trim sequenced reads. Reads with length >25 or <21 bp were removed. Finally, cleaned 24-nt reads were mapped to the cultivated octoploid strawberry (*F. × ananassa*) genome [11] by bowtie2 and identified to siRNA with Shortstack [39].

### RT–qPCR assays

Total RNA was extracted from strawberry fruits through a CTAB-based method referring to the published description [40]. Reverse transcription was carried out with 1000 g RNA treated with DNase, and the resulting cDNA products were diluted 20-fold for further gene cloning and RT–qPCR. An RT–qPCR reaction was carried out using SsoFast EvaGreen Supermix (Bio-Rad, USA) referring to the protocol. The specificity of RT–qPCR primers was checked via the melting curve, product sequencing, and agarose gel electrophoresis. Two internal reference genes, *FaRIB413* (gene33863) and *GAPDH* (AB363963.1), were used to normalize target gene expression by the 2^-ΔΔCT^ method [41]. The gene expression level was normalized against the geometric mean of *FaRIB413* and *GAPDH*. The RT–qPCR assay was carried out with three biological repetitions. The specific primers in the RT–qPCR assay are listed in Supplementary Data Table S3.

### Temperature treatment and 5-azacytidine treatment

For temperature treatment, postharvest ‘Yuexin’ strawberry fruits at T stage of similar size were used. The collected fruits were stored in two identical freezers at 10 and 20°C, respectively. Fruits were sampled at 1, 2, 3, 4, and 5 days after treatment. Each biological replicate included four fruits and three biological repetitions were sampled. ‘Yuexin’ fruits at the green (G) stage were used for 5-azacytidine treatment. Previous studies used 20 mM 5-azacytidine (Sigma) dissolved in ddH_2_O that was directly sprayed on the fruits [17]. In this study, 1 mM 5-azacytidine was injected into the whole strawberry fruit. About 2 ml of 5-azacytidine was injected into each fruit until the whole fruit became hydrophanous. On the 5th day after the first injection, the second injection was performed. Fruits remained on the plant and continued to grow. Fruits were collected on days 0, 4, 6, 8, and 11 after the first injection. 5-Azacytidine treatment was carried out between 14 December 2019 and 25 December 2019. The temperature in the tunnel greenhouse covered with plastic film was 15.20 ± 4.40°C.

### Detection of DNA methylation within the FaQR promoter

Genomic DNA of strawberry fruits was extracted through a CTAB-based method, then samples were treated with bisulfite [42]. PCR amplification was conducted using the bisulfite-treated DNA with specific primers targeting different regions located within the *FaQR* promoter and gene sequence. Then, purified PCR products were inserted into the pGEM-T Easy vector (Promega). At least 12 single colonies in each PCR amplification were sequenced and three biological replicates were performed. The DNA methylation pattern of the *FaQR3* promoter was analyzed using Kismeth [43] and CyMATE [44]. Primers for detecting DNA methylation in promoter and gene are listed in Supplementary Data Table S4.

### Dual-luciferase assays

Tobacco showed high efficiency for transient overexpression, while strawberry fruit exhibited low efficiency. Thus, tobacco is recognized as a stable system for the dual-luciferase assay, and is widely used for detecting the effect of TFs on their targets [45]. Referring to the published protocol [36], the full coding sequences of TFs were recombined into the pGreen II 0029 62-SK vector, and 1910 and 1418 bp of the *FaQR3* promoter (*FaQR3Pro*) was recombined into the pGreen II 0800-LUC vector. *Renilla* luciferase (REN) driven by a 35S promoter in pGreen II 0800-LUC vector served as an internal control. Tobacco plants (*Nicotiana benthamiana*) grown in a growth chamber at 24°C with a light/dark cycle of 16 h/8 h were used for dual-luciferase assays. A mixture of 1 ml *A. tumefaciens* (strain GV3101) suspension containing TFs-SK plasmid and 100 μl *A. tumefaciens* suspension harboring a promoter-LUC plasmid was co-infiltrated into 4-week-old tobacco leaves through a needleless syringe. The *A. tumefaciens* suspension was adjusted to the working concentration (OD_600_ = 0.75) with infiltration buffer. The enzyme activities were detected on the third day after injection using dual-luciferase reagents (Promega) by a Modulus Luminometer (Promega). The absolute LUC/REN value of the empty SK vector on the *FaQR3* promoter was identified as 1, and served as a control. At least eight biological repetitions were performed for each TF–promoter interaction assay.

### Transient overexpression and RNAi in strawberry fruit

The pGreen II 0029 62-SK vector harboring *FaAGO4* was used for the *FaAGO4* transient overexpression assay in strawberry fruits. The pHellsgate vector was used for transient RNAi of *FaAGO4*, *FaDML1*, and *FaDML2*. Forward and reverse cDNA fragments of each gene produced by PCR amplification were inserted into the pHellsgate vector by gateway recombination technology (Invitrogen) to produce the *FaAGO4*-RNAi, *FaDML1*-RNAi, and *FaDML2*-RNAi construct, respectively. The primers used in PCR amplification are shown in Supplementary Data Table S5. The resulting constructs were independently transformed into *A. tumefaciens* (strain GV3101). The *A. tumefaciens* suspension was adjusted to the working concentration (OD_600_ = 1) with infiltration buffer. Twelve fruits of similar size were selected for agroinfiltration, and ~2 ml *A. tumefaciens* suspension was injected by needleless syringe. *Agrobacterium tumefaciens* suspension was evenly infiltrated into the basal section of strawberry fruits at G stage until the whole strawberry became hydrophanous. After infiltration, strawberries were left attached to the plants until harvest. After 12 days of injection, each strawberry fruit was sampled as an individual biological repetition. Fruits infiltrated with *A. tumefaciens* containing an empty pGreen II 0029 62-SK vector or empty pHellsgate vector under the same infiltration conditions served as a control treatment. Transient expression was performed between 25 November 2020 and 13 December 2020, and the temperature in the tunnel greenhouse covered with plastic film was ~17.51 ± 4.05°C.

### Statistical analysis

Figures were plotted through Sigmaplot 12.0 and GraphPad Prism 8.3.0 [46]. Linear regressive analysis was performed using SPSS Statistics 22.0. Heat map construction was carried out using MultiExperiment Viewer 4.8.1. Significant differences were performed using one-way ANOVA by SPSS Statistics 22.0. Least significant differences were assessed at 0.05 using SPSS Statistics software 22.0.

## Acknowledgements

We thank Jiangsu Academy of Agricultural Sciences for providing the leaves of ‘Camarosa’. This work was supported by the National Key Research and Development Program of China (2022YFD2100100), the National Natural Science Foundation of China (32002004), the 111 Project (B17039), and Fundamental Research Funds for the Central Universities.

## Author contributions

Y. (Yunduan) L. performed most of the experiments and data analysis, and wrote the manuscript. J.L. and W.C. performed small-RNA analysis. Y. (Yichen) L., Y.S. (Sun) and Y.Z. contributed part of the experiments. Y.S. (Shi), Z.L., and K.C. provided guidance for the experiment and revised the manuscript. D.G. edited and polished the manuscript. X.Y. and G.J. created the new cultivar ‘Yuexin’ and performed field management. All authors read and approved the final manuscript.

## Data availability

The datasets supporting the conclusions of this article are included in the article and additional files. The GenBank accession number of *FaQR* is AY158836.1 [7]. The accession numbers of *AP2/ERF* genes in strawberry referred to previous studies [10]. Accession numbers of the remaining genes are shown in Supplementary Data Table S2.

## Conflict of interest

None declared.

## Supplementary data

Supplementary data is available at *Horticulture Research* online.

## Supplementary Material

Web_Material_uhad131
