# Supplementary material for: DNA methylation mediated by RdDM pathway and demethylation affects furanone accumulation through regulation of QUINONE OXIDOREDUCTASE in strawberry
Source: Hortic Res. 2023 Jul 11;10(8):uhad131. doi: 10.1093/hr/uhad131 (PMC10407599; doi:10.1093/hr/uhad131)
Supplement: Web_Material_uhad131 [file web_material_uhad131.zip › Supplementary Information 19-5-2023.pdf]

DNA methylation mediated by RdDM pathway and demethylation affects furanone accumulation through regulation of *QUINONE OXIDOREDUCTASE* in strawberry

Yunduan Li[1†], Yanna Shi[1, 2, 3†], Yichen Li[1], Jiao Lu[1], Yunfan Sun[1], Yuanyuan Zhang[1], Wenbo Chen[1, 2, 3], Xiaofang Yang[4], Donald Grierson[1, 5], Zhaobo Lang[6*], Guihua Jiang[4*], Kunsong Chen[1, 2, 3*]

[1]College of Agriculture & Biotechnology, Zhejiang University, Zijingang Campus, Hangzhou 310021, Zhejiang, China

[2]Zhejiang Provincial Key Laboratory of Horticultural Plant Integrative Biology, Zhejiang University, Zijingang Campus, Hangzhou 310021, Zhejiang, China

[3]The State Agriculture Ministry Laboratory of Horticultural Plant Growth, Development and Quality Improvement, Zhejiang University, Zijingang Campus, Hangzhou 310021, Zhejiang, China

[4]Institute of Horticulture, Zhejiang Academy of Agricultural Sciences, Hangzhou 310021, Zhejiang, China

[5]Division of Plant and Crop Sciences, School of Biosciences, University of Nottingham, Sutton Bonington Campus, Loughborough LE12 5RD, United Kingdom

[6]National Key Laboratory of Plant Molecular Genetics, CAS Center of Excellence in Molecular Plant Sciences, Institute of Plant Physiology and Ecology, Shanghai Institutes for Biological Sciences, Chinese Academy of Sciences, Shanghai 200032, China

[†]These authors contributed equally to this manuscript

*Corresponding authors

Fig. S1. Promoters of four *FaQR* homologs in 'Yuexin' cultivated strawberry. Sequence alignment was performed by DNAMAN software.

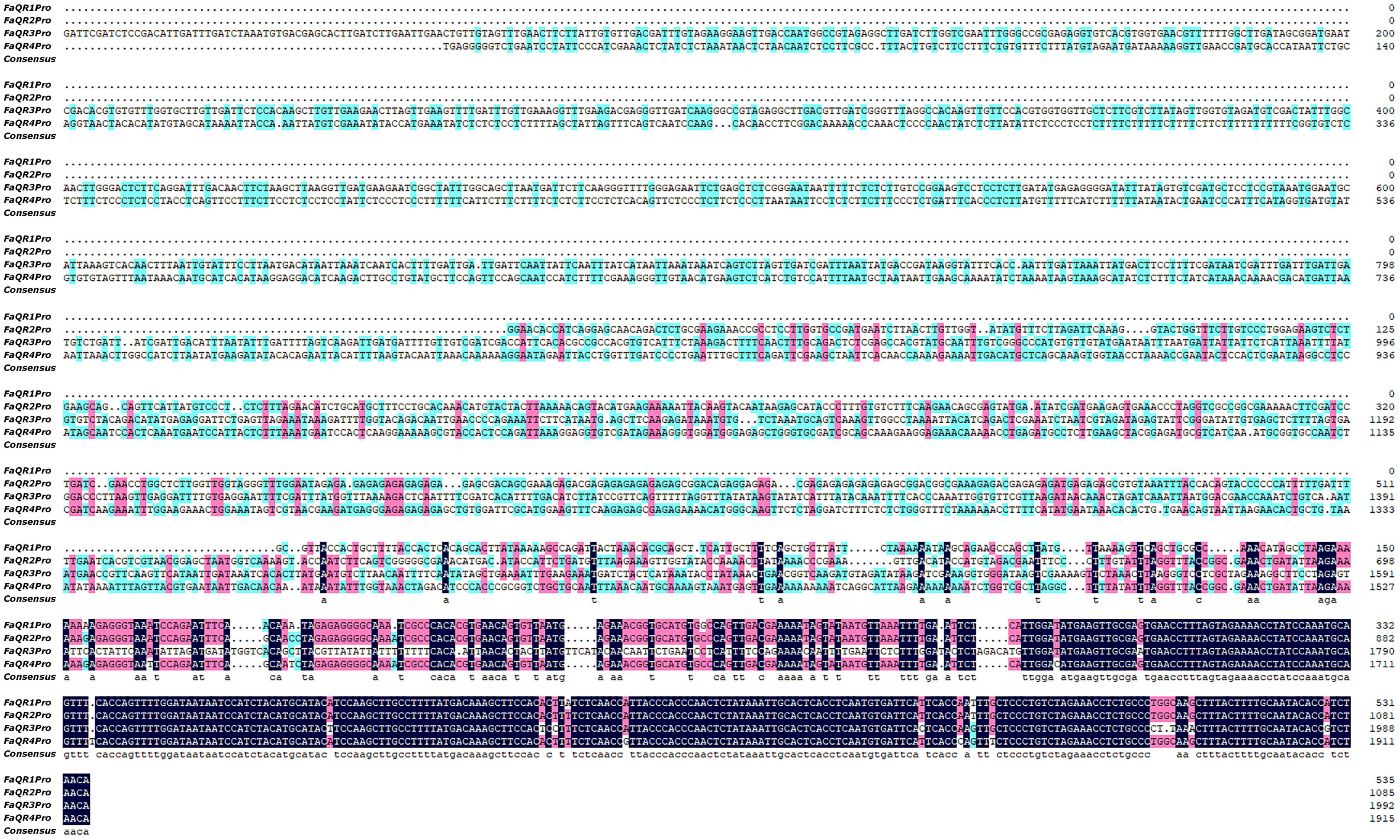

Fig. S2. The sequences of four *FaQR* homologs in 'Yuexin' cultivated strawberry. The *FvQR* coding sequence is from *F. vesca*. Sequence alignment was performed by DNAMAN software. Primers used in Fig. S4d were designed in the consistent regions of these four homologs (the red lines).

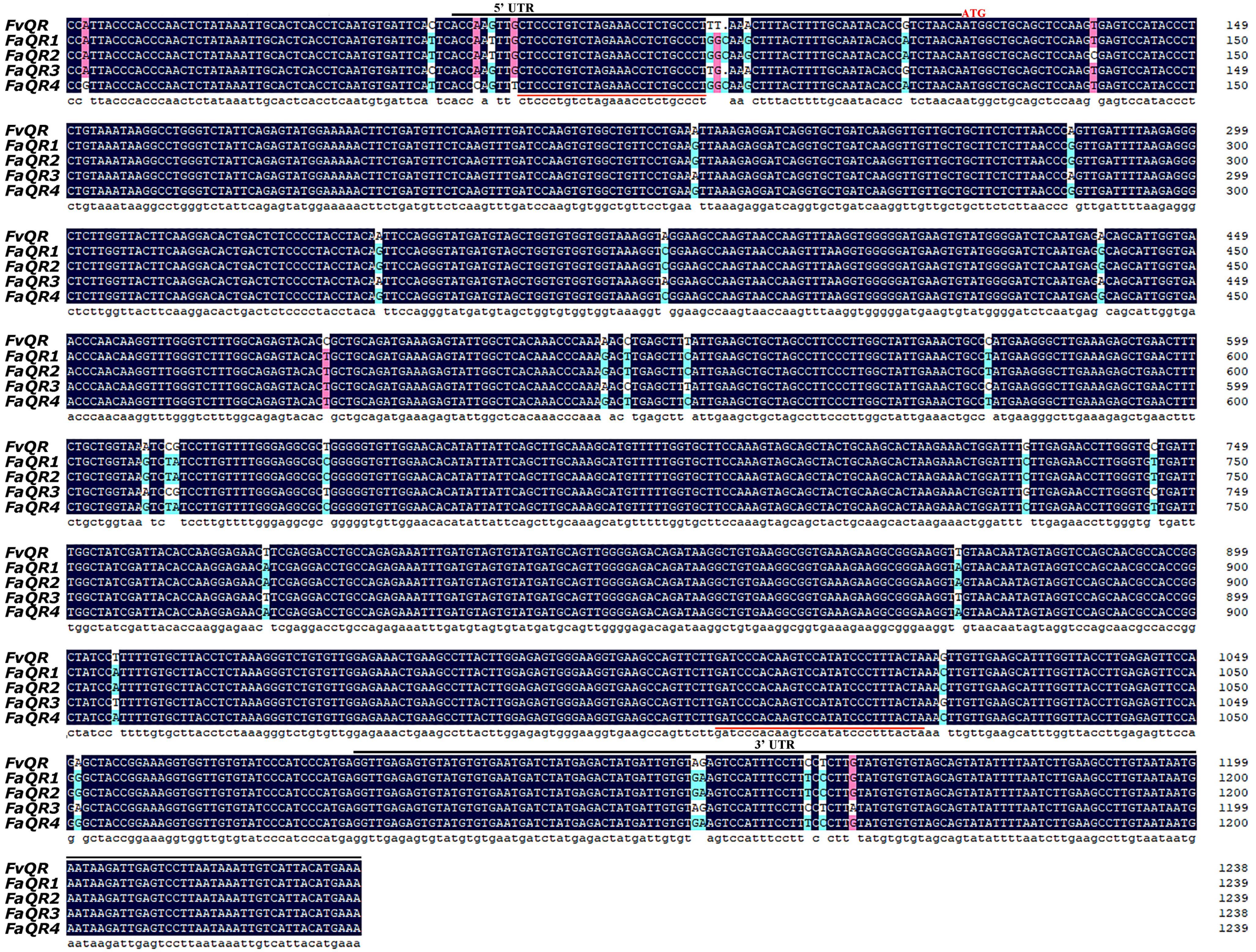

Fig. S3. The amino acid sequences of four *FaQR* homologs in 'Yuexin' cultivated strawberry. The amino acid sequence of *FvQR* are from *F. vesca*. Sequence alignment was performed by DNAMAN software.

```
FvQR      MAAAPSESIPSVNKAWVYSEYGKTSDVLKFDPSVAVPEIKEDQVLIKVVAASLNPVDFKRALGYFKDTDSPLPTIPGYDVAGVVVKVGSQVTKFKVGDEV    100
FaQR1     MAAAPSESIPSVNKAWVYSEYGKTSDVLKFDPSVAVPEVKEDQVLIKVVAASLNPVDFKRALGYFKDTDSPLPTVPGYDVAGVVVKVGSQVTKFKVGDEV    100
FaQR2     MAAAPSESIPSVNKAWVYSEYGKTSDVLKFDPSVAVPEVKEDQVLIKVVAASLNPVDFKRALGYFKDTDSPLPTVPGYDVAGVVVKVGSQVTKFKVGDEV    100
FaQR3     MAAAPSESIPSVNKAWVYSEYGKTSDVLKFDPSVAVPEIKEDQVLIKVVAASLNPVDFKRALGYFKDTDSPLPTIPGYDVAGVVVKVGSQVTKFKVGDEV    100
FaQR4     MAAAPSESIPSVNKAWVYSEYGKTSDVLKFDPSVAVPEVKEDQVLIKVVAASLNPVDFKRALGYFKDTDSPLPTVPGYDVAGVVVKVGSQVTKFKVGDEV    100
Consensumaaapsesipsvnkawvyseygktsdvlkfdpsvavpe kedqvlikvvaaslnpvdfkralgyfkdtdsplpt pgydvagvvvkvgsqvtkfkvgdev

FvQR      YGDLNETALVNPTRFGSLAEYTAADERVLAHKPKNLSFIEAASLPLAIETAHEGLERAELSAGKSVLVLGGAGGVGTHIIQLAKHVFGASKVAATASTKK    200
FaQR1     YGDLNEAALVNPTRFGSLAEYTAADERVLAHKPKDLSFIEAASLPLAIETAYEGLERAELSAGKSILVLGGAGGVGTHIIQLAKHVFGASKVAATASTKK    200
FaQR2     YGDLNEAALVNPTRFGSLAEYTAADERVLAHKPKDLSFIEAASLPLAIETAYEGLERAELSAGKSILVLGGAGGVGTHIIQLAKHVFGASKVAATASTKK    200
FaQR3     YGDLNETALVNPTRFGSLAEYTAADERVLAHKPKNLSFIEAASLPLAIETAHEGLERAELSAGKSVLVLGGAGGVGTHIIQLAKHVFGASKVAATASTKK    200
FaQR4     YGDLNEAALVNPTRFGSLAEYTAADERVLAHKPKDLSFIEAASLPLAIETAYEGLERAELSAGKSILVLGGAGGVGTHIIQLAKHVFGASKVAATASTKK    200
Consensuygdlne alvnptrfgslaeytaadervlahkpk lsfieaaslplaieta egleraelsagks lvlggaggvgthiiqlakhvfgaskvaatastkk

FvQR      LDLLRTLGADLAIDYTKENFEDLPEKFDVVYDAVGETDKAVKAVKEGGKVVTIVGPATPPAILFVLTSKGSVLEKLKPYLESGKVKPVLDPTSPYPFTKV    300
FaQR1     LDFLRTLGVDLAIDYTKENIEDLPEKFDVVYDAVGETDKAVKAVKEGGKVVTIVGPATPPAIHFVLTSKGSVLEKLKPYLESGKVKPVLDPTSPYPFTKL    300
FaQR2     LDFLRTLGVDLAIDYTKENIEDLPEKFDVVYDAVGETDKAVKAVKEGGKVVTIVGPATPPAIHFVLTSKGSVLEKLKPYLESGKVKPVLDPTSPYPFTKL    300
FaQR3     LDLLRTLGADLAIDYTKENFEDLPEKFDVVYDAVGETDKAVKAVKEGGKVVTIVGPATPPAILFVLTSKGSVLEKLKPYLESGKVKPVLDPTSPYPFTKV    300
FaQR4     LDFLRTLGVDLAIDYTKENIEDLPEKFDVVYDAVGETDKAVKAVKEGGKVVTIVGPATPPAIHFVLTSKGSVLEKLKPYLESGKVKPVLDPTSPYPFTKL    300
Consensuld lrtlg dlaidytken edlpekfdvvydavgetdkavkavkeggkvvtivgpatppai fvltskgsvleklkpylesgkvkpvldptspypftk

FvQR      VEAFGYLESSRATGKVVVYPIP    322
FaQR1     VEAFGYLESSRATGKVVVYPIP    322
FaQR2     VEAFGYLESSRATGKVVVYPIP    322
FaQR3     VEAFGYLESSRATGKVVVYPIP    322
FaQR4     VEAFGYLESSRATGKVVVYPIP    322
Consensuveafgylessratgkvvvypip
```

Fig. S4. The expression levels of four *FaQR* homologs distinguished by RT-qPCR and the proportion of *FaQR* homologs distinguished by sequencing single colonies. **(a)** Specific primers of *FaQR3* and *FaQR1/2/4* used in RT-qPCR. **(b)** Sequencing results of RT-qPCR products. **(c)** Sequencing results of single colonies in RT-qPCR assays. The RT-qPCR product amplified by specific primers of *FaQR3* and *FaQR1/2/4* was cloned to pGEM-T Easy vector (Promega), and transformed into *E.coli* DH-5α (TaKaRa). (**d**) The percentage of four *FaQR* homologs was determined by cloning. Primers were designed in the consistent regions of these four homologs (the red lines in Fig. S2). PCR amplification was performed from the cDNA of 'Yuexin' fruits at four ripening stages. The PCR products were cloned to pGEM-T Easy vector (Promega) and transformed into *E.coli* DH-5α (TaKaRa). A total of 50 single colonies were sequenced in each stage. The frequency of these four homologs in 50 single colonies were analyzed. G, T, IR and R represent green, turning, intermediate red and full red stage, respectively. The proportion of each *FaQR* at each stage can be compared, and the data from different stages cannot be compared. There was one base difference (Fig. S2 and Fig. S4d) between *FaQR2* and other *FaQR* homologs at 18 bp downstream of ATG, which was used to distinguish *FaQR2* in sequencing results.

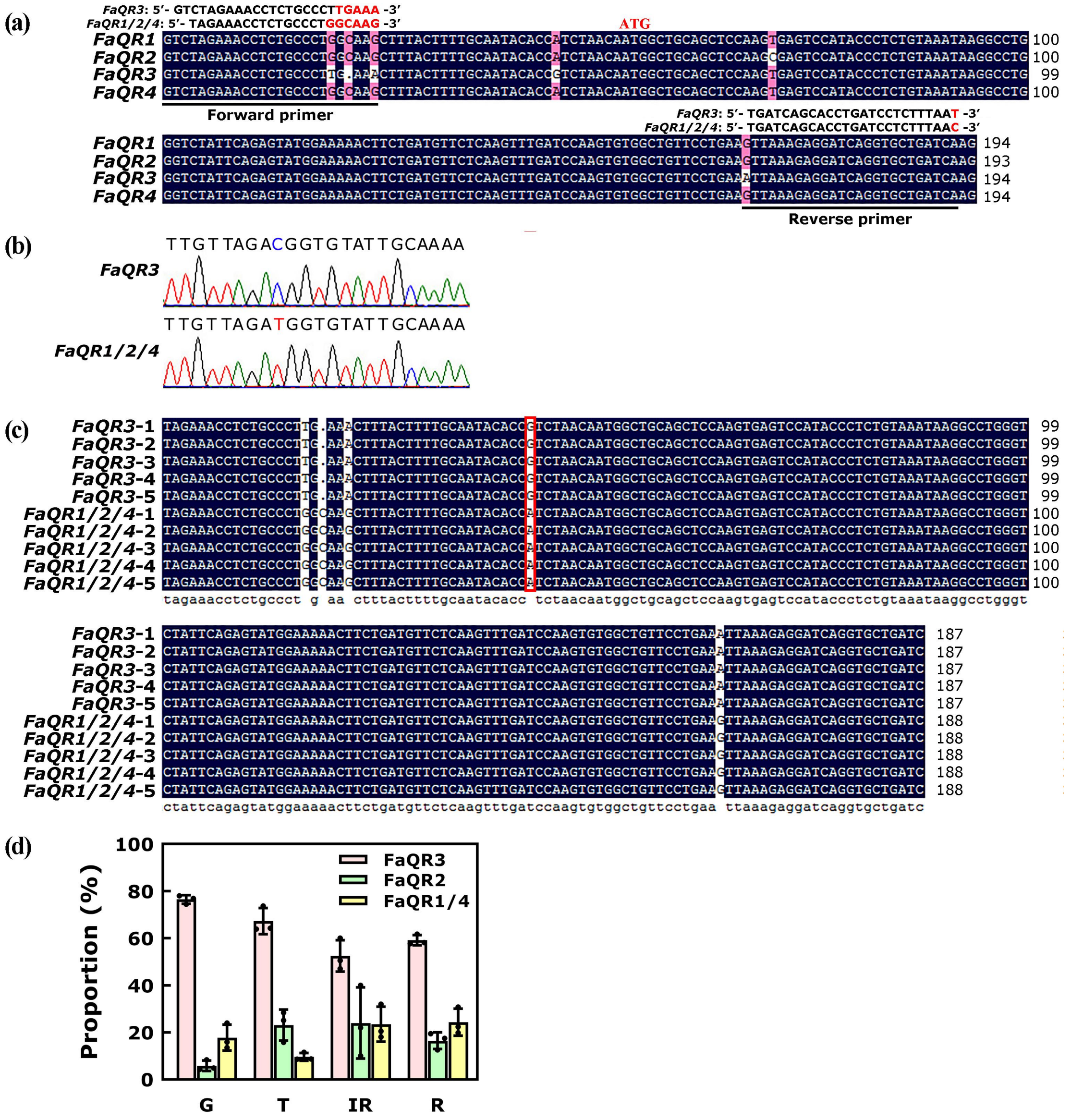

Fig. S5. DNA methylation levels of FaQR3Pro-M2 during fruit ripening in strawberry (*Fragaria × ananassa* Duch. cv. Hongjia). The original data were derived from previously published whole genome bisulfite sequencing (Cheng et al., 2018). The whole fruit was used for sequencing. The cleaned reads were aligned to 'Camarosa' (*Fragaria × ananassa*) genome. Asterisks represent significant differences using one-way ANOVA (*, 0.01 < p < 0.05; **, P < 0.01). Fa1, G stage; Fa2, IR stage; Fa3, R stage.

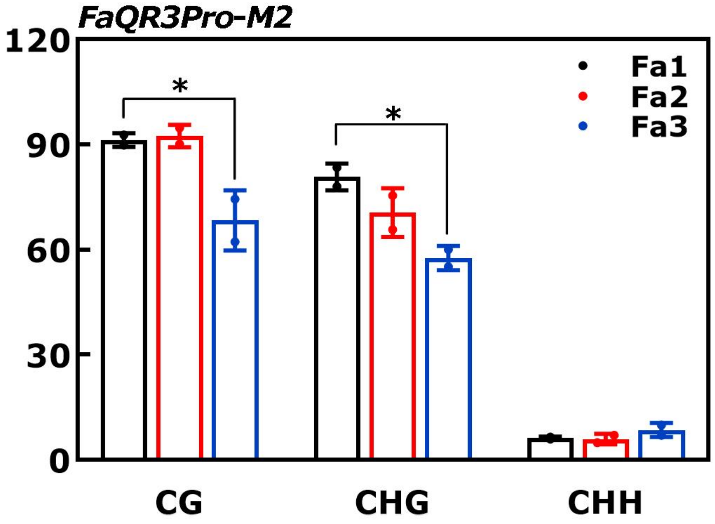

Fig. S6. Changes of DNA methylation in FaQR3Pro-M2, *FaQR3* expression and furanone content during fruit ripening in *F. Vesca* and different cultivated octoploid strawberries. **(a)** DNA methylation levels of FaQR3Pro-M2. **(b)** *FaQR3* expression. **(c)** Furanone content. FW, fresh weight. Bars stand for standard deviation from three biological replicates. Asterisks represent significant differences using one-way ANOVA (*, 0.01 < p < 0.05; **, P < 0.01). When analyzing the significant differences, the DNA methylation level of promoter, *FaQR3* expression and furanone content at each stage was compared with that in G stage.

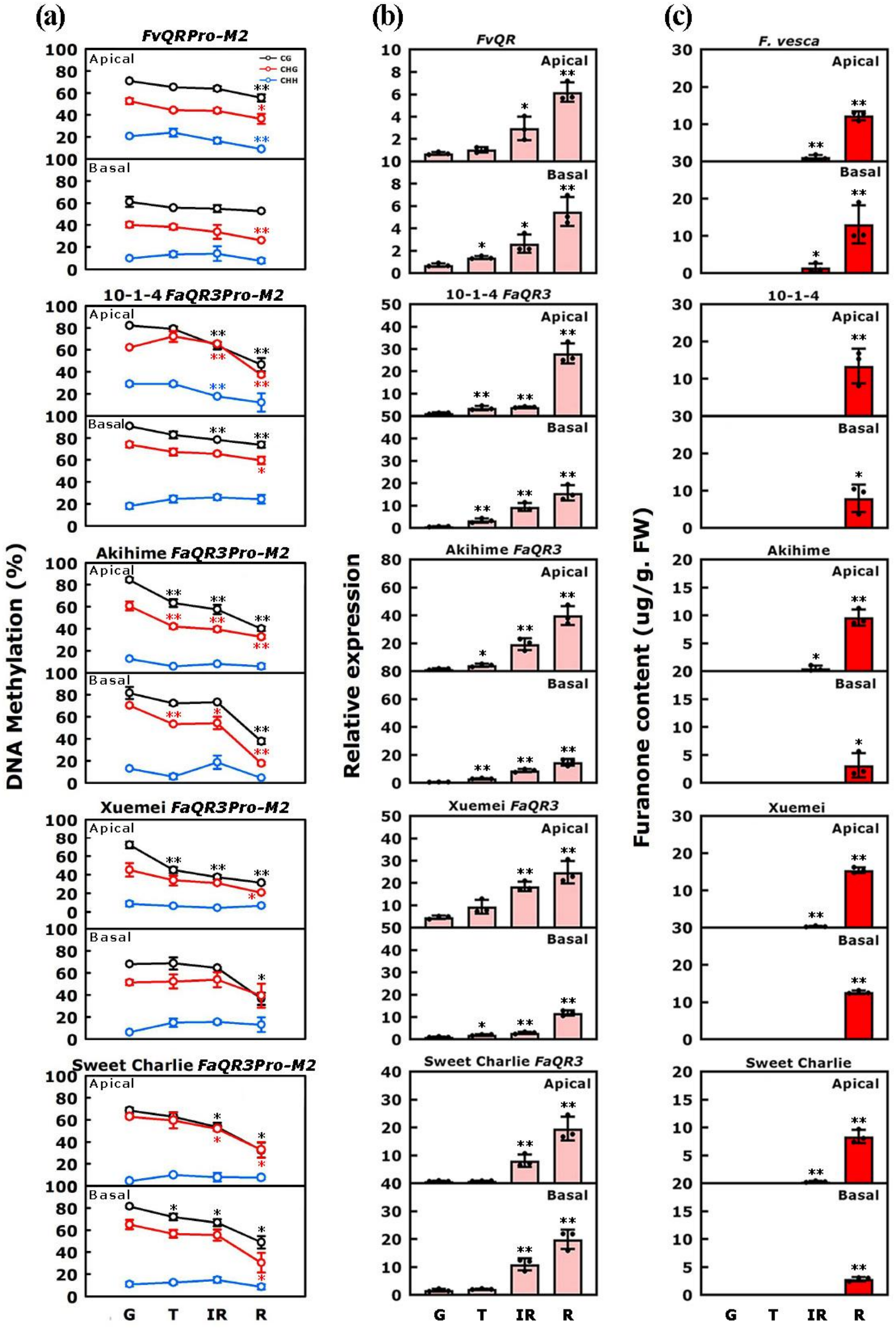

Fig. S7. Development and ripening stages of *F. vesca* and different cultivated strawberries.

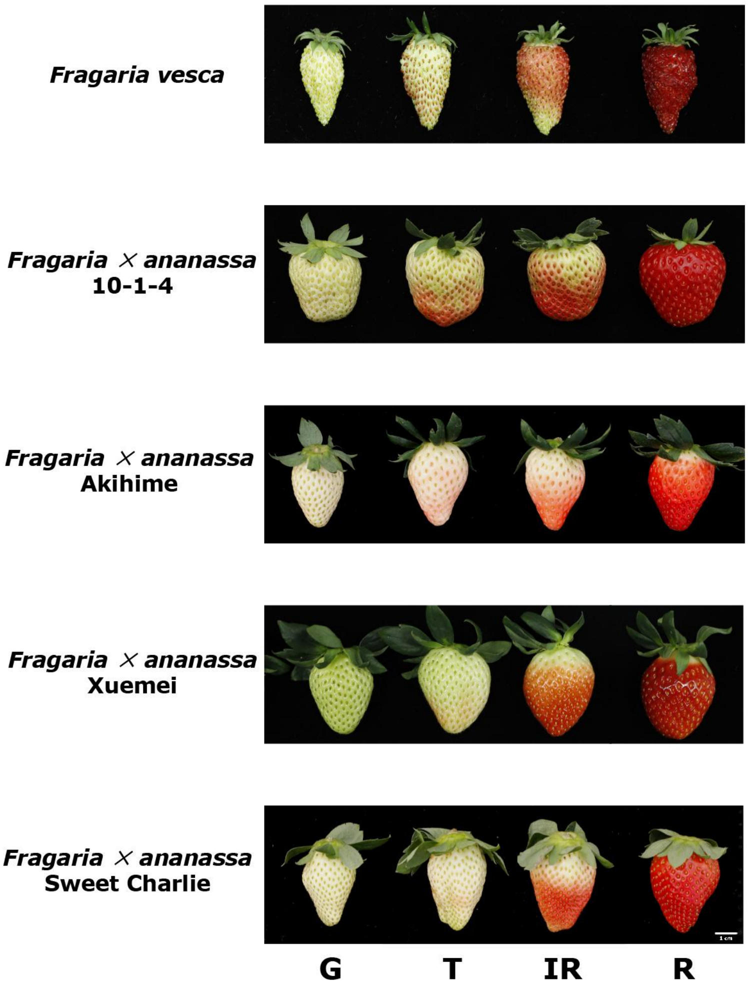

Fig. S8. Analysis of 21-nt, 22-nt and 24-nt siRNA in FaQR3Pro at single-base resolution. **(a)** Integrated Genome Browser (IGB) display of DNA methylation levels of FaQR3Pro and enrichment of 21-nt, 22-nt and 24-nt siRNA in FaQR3Pro. DNA methylation levels of cytosines are indicated by the heights of the vertical bars on each track. Cleaned 24-nt reads were mapped to cultivated octoploid strawberry (*Fragaria* × *ananassa*) genome. The data used for analysis were from fruits at G and R stage, which corresponds to Fa1 and Fa3 stage in the previous study, respectively (Cheng et al., 2018). The region on the horizontal straight line represents siRNAs in FaQR3Pro at Fa1 stage. The region below the horizontal straight line represents siRNAs in FaQR3Pro at Fa3 stage. Each shadowbox represent one single read of a specific 24-nt siRNA. **(b)** Enriched levels of 21-nt, 22-nt and 24-nt siRNA in FaQR3Pro. The mapped reads of siRNAs were normalized by total cleaned reads. Asterisks represent significant differences using one-way ANOVA (*, 0.01 < p < 0.05; **, P < 0.01).

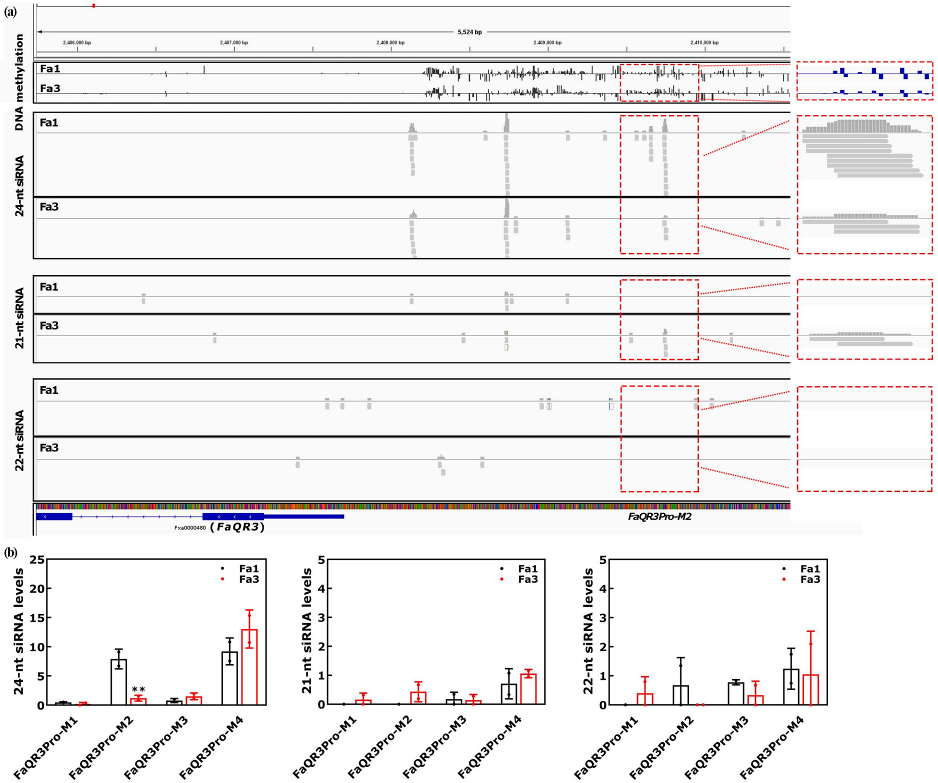

Fig. S9. The phylogenetic tree of the DML and DME proteins in strawberry, tomato and *Arabidopsis thaliana*. The evolutionary tree was constructed by MEGA 6.0 using neighbour-joining (NJ) method. The bootstrap analysis was carried out with 1000 replicates and p-distance, and full-length protein sequences were used.

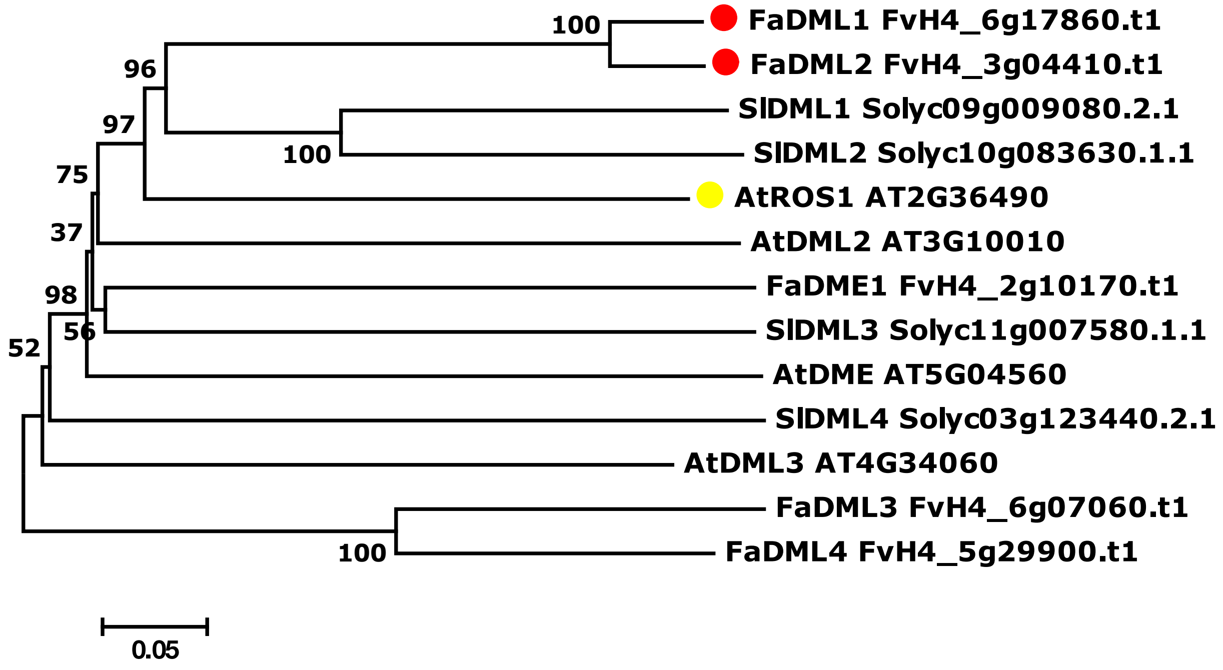

Fig. S10. Regulatory effects of TFs on the FaQR3Pro with or without FaQR3Pro-M2 fragment by dual-luciferase assays. **(a)** Analysis of conserved cis-element motifs in FaQR3Pro. Symbols with different shapes represent different cis-acting elements. **(b)** Transient activity analysis of the FaQR3Pro with or without FaQR3Pro-M2 fragment by dual-luciferase assays. SK refers to empty pGreenII 0029 62-SK vector and is set as 1. Error bars represent standard deviation based on at least eight biological replicates. Asterisks represent significant differences using one-way ANOVA (*, $0.01 < p < 0.05$; **, $P < 0.01$).

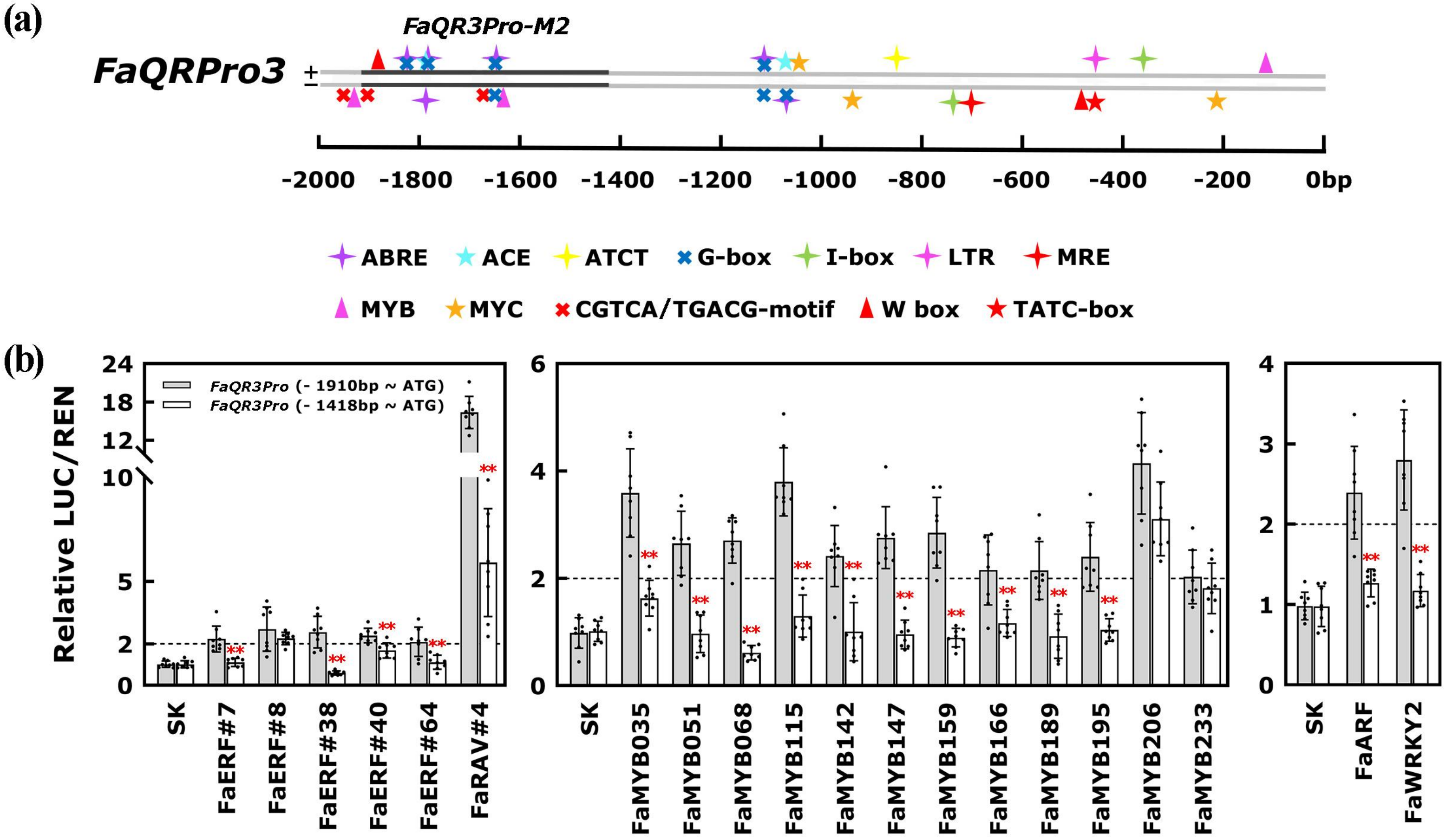

Fig. S11. Regulatory effects of TFs on FaQR3Pro by dual-luciferase assays. SK refers to empty pGreenII 0029 62-SK vector and is set as 1. Error bars represent standard deviation based on at least eight biological replicates.

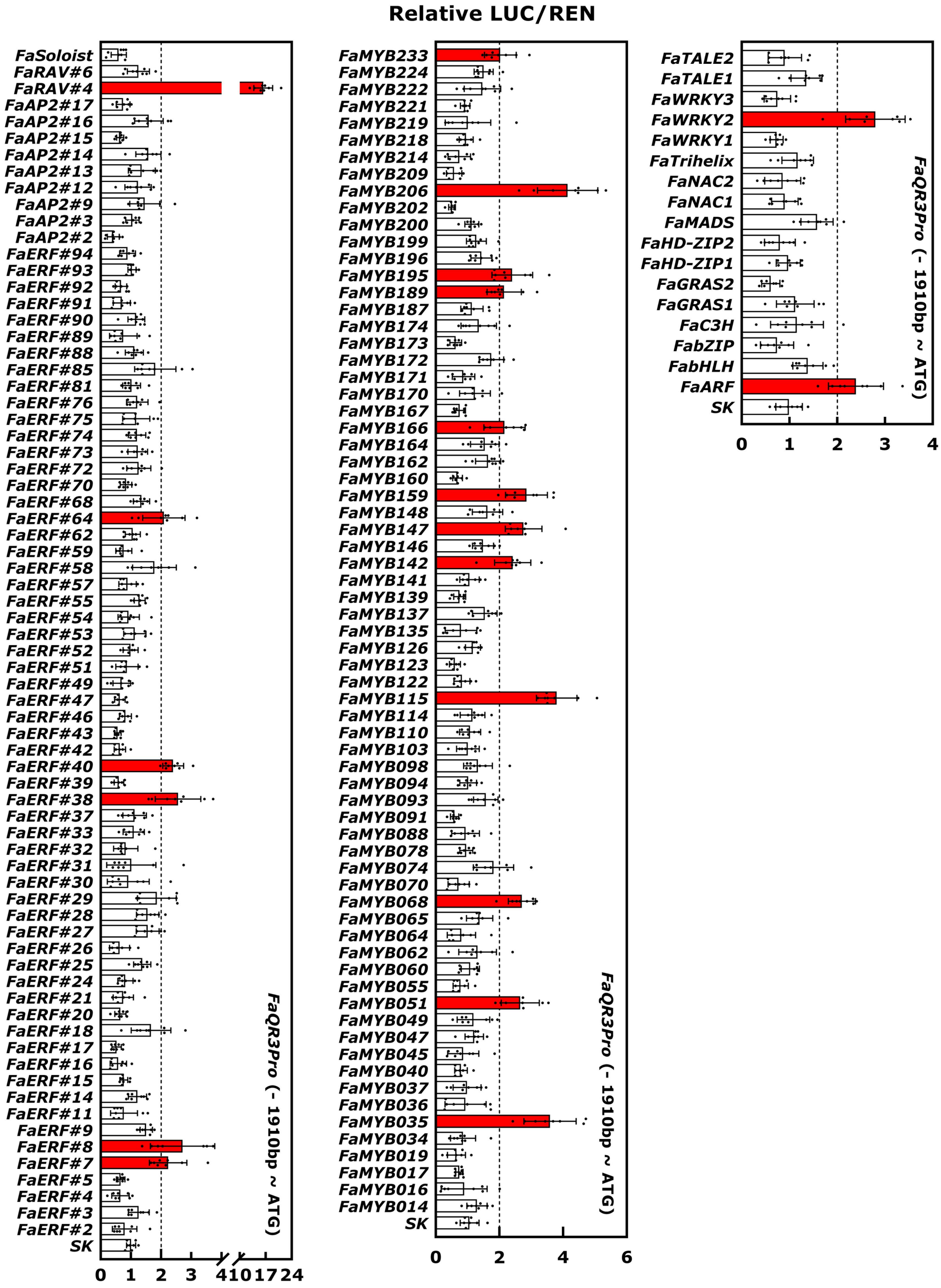

Fig. S12. DNA methylation levels of FaQR1Pro, FaQR2Pro and FaQR4Pro, and *FaQR1/2/4* expression at 10℃ and 20℃ treatment. **(a)** The relative expression of *FaQR1/2/4*. **(b)** DNA methylation levels of FaQR1Pro, FaQR2Pro and FaQR4Pro. Bars stand for standard deviation from three biological replicates. Asterisks represent significant differences using one-way ANOVA (*, $0.01 < p < 0.05$; **, $P < 0.01$).

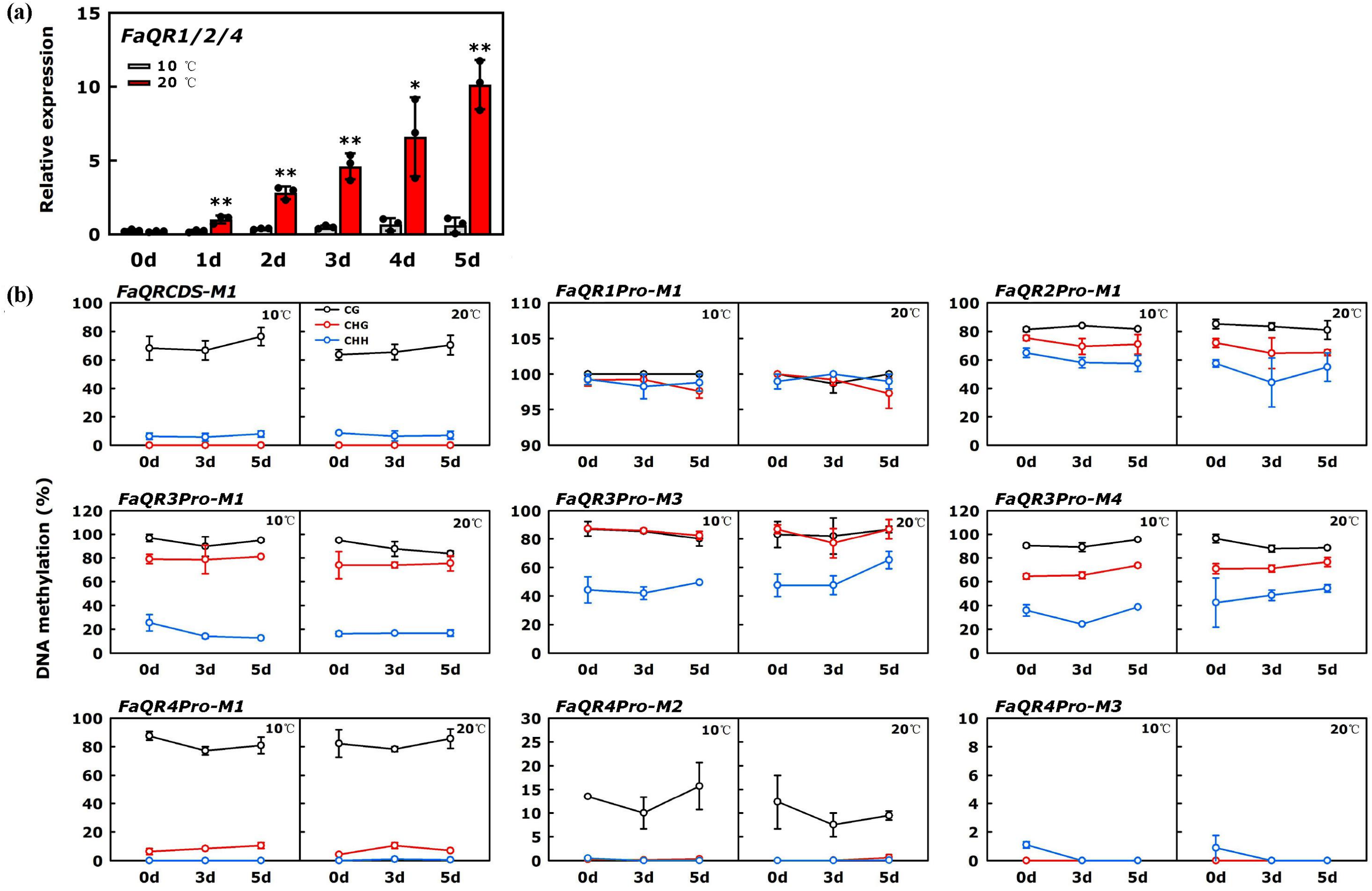

Fig. S13. A potential regulatory model of the effect of DNA methylation modification in regulating furanone biosynthesis in strawberry fruit. RdDM pathway and demethylation pathway are involved in the DNA methylation modification in the *FaQR3* promoter during strawberry fruit ripening. The down-regulated DNA methylation within the *FaQR3* promoter contributes to *FaQR3* expression and furanone accumulation.

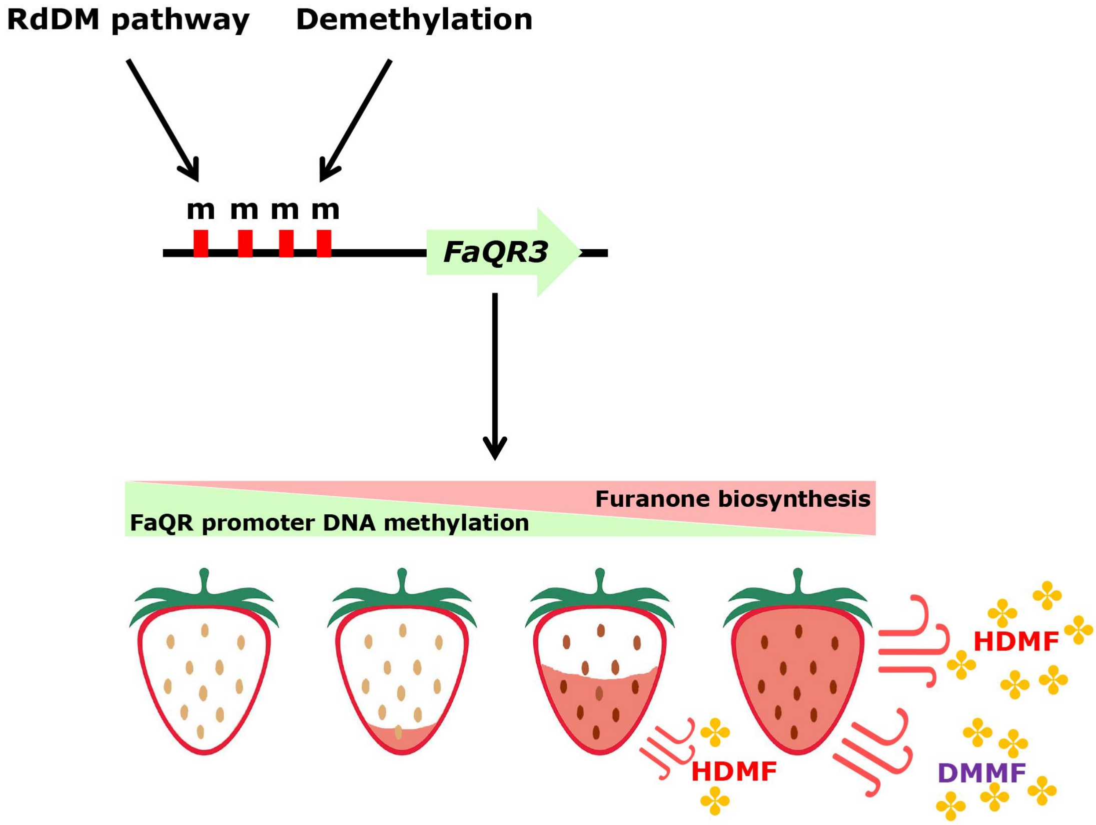

Table S1. The promoter of four *FaQR* homologs were found in many octoploid strawberries (*Fragaria* × *ananassa*). Specific forward primers were designed according to each *FaQR* promoter sequence for PCR analysis. Reverse primer were designed in the same region of *FaQR* homologs near the 3' UTR. PCR products were purified and cloned into the pGEM-T Easy vector (Promega), and then single colony was sequenced. + represent existence of the promoter region of *FaQR* homologs in the corresponding cultivar.

	FaQR1Pro	***FaQR2Pro***	***FaQR3Pro***	***FaQR4Pro***
Camarosa		+	+	+
10-1-4	+	+	+	+
Sweet Charlie	+	+	+	+
Amaou	+	+	+	+
Akihime	+	+	+	+
Xuemei	+	+	+	+
Mengxiang	+	+	+	+
Darselect	+	+	+	
Yuexin	+	+	+	+

Table S2. Accession numbers of all genes used in this study.

Gene name	Accession number	GenBank number
FaQR	gene28406	AY158836.1
FaRIB413	gene33863	
FaGAPDH	gene07104	AB363963.1
FaAGO4	gene07657/FvH4_3g27260.t	
SlDML1	Solyc09g009080.2.1	
SlDML2	Solyc10g083630.1.1	
SlDML3	Solyc11g007580.1.1	
SlDML4	Solyc03g123440.2.1	
FaDML1	gene30143/FvH4_6g17860.t	
FaDML2	gene30462/FvH4_3g04410.t	
FaDML3	gene31547/FvH4_6g07060.t	
FaDML4	gene11785/FvH4_5g29900.t	
FaDME1	gene01635/FvH4_2g10170.t	
AtROS1	AT2G36490	AAP37178.1
AtDME	AT5G04560	AED90760.1
AtDML2	AT3G10010	
AtDML3	AT4G34060	
FaMYB014	gene23919	XP_011463172.1
FaMYB016	gene24027	XP_004287994.1
FaMYB017	gene24094	XP_004288067.1
FaMYB019	gene05764	XP_004288258.1
FaMYB034	gene25445	XP_011457840.1
FaMYB035	gene07646	XP_004289866.1
FaMYB036	gene08084	XP_004289939.1
FaMYB037	gene08317	XP_004289982.1
FaMYB040	gene17613	XP_004292373.1
FaMYB045	gene11231	XP_011458806.1
FaMYB047	gene02652	XP_011458889.1
FaMYB049	gene00185	XP_004291065.1
FaMYB051	gene08793	XP_004291202.2
FaMYB055	gene21456	XP_004291570.1
FaMYB060	gene19598	XP_004295264.1
FaMYB062	gene19812	XP_004293386.1
FaMYB064	gene19866	XP_011460083.1
FaMYB065	gene19867	XP_004293430.1
FaMYB068	gene01998	XP_004293474.1
FaMYB070	gene30736	XP_004293617.1
FaMYB074	gene29666	XP_004293945.1
FaMYB078	gene07984	XP_004294268.1
FaMYB088	gene24821	XP_004295005.1
FaMYB091	gene05178	XP_004295112.1
FaMYB093	gene27918	XP_004136602
FaMYB094	gene11462	XP_004296369.1
FaMYB098	gene03865	XP_004297138.1

FaMYB103	gene04057	XP_004297358.1
FaMYB110	gene00738	XP_004297686.2
FaMYB114	gene32264	XP_004298675.2
FaMYB115	gene32268	XP_004298679.1
FaMYB122	gene03712	XP_004299054.1
FaMYB123	gene20434	XP_004299098.1
FaMYB126	gene26214	XP_004299236.1
FaMYB135	gene09331	XP_011464470.1
FaMYB137	gene09407	XP_004299542.1
FaMYB139	gene09510	XP_004299599.2
FaMYB141	gene12179	XP_004299744.1
FaMYB142	gene25685	XP_004299892.1
FaMYB146	gene13743	XP_004300205.1
FaMYB147	gene10484	XP_004300274.1
FaMYB148	gene10772	XP_004300321.1
FaMYB159	gene31413	XP_004302217.1
FaMYB160	gene22961	XP_004302323.1
FaMYB162	gene22515	XP_004302418.1
FaMYB164	gene13452	XP_004302587.1
FaMYB166	gene18815	XP_004302668.1
FaMYB167	gene18756	XP_004302679.1
FaMYB170	gene18104	XP_004302837.1
FaMYB171	gene25733	XP_004302861.1
FaMYB172	gene05956	XP_004302915.1
FaMYB173	gene30156	XP_004305362.1
FaMYB174	gene17673	XP_004303133.1
FaMYB187	gene07405	XP_011467416.1
FaMYB189	gene07418	XP_004305745.1
FaMYB195	gene26289	XP_004305954.1
FaMYB196	gene10329	XP_004304144.1
FaMYB199	gene03817	XP_004304297.1
FaMYB200	gene22621	XP_004304372.1
FaMYB202	gene09621	XP_004304454.1
FaMYB206	gene28443	XP_004306352.1
FaMYB209	gene26789	XP_004306497.2
FaMYB214	gene19228	XP_004306739.1
FaMYB218	gene23544	XP_011469263.1
FaMYB219	gene14244	XP_004307092.1
FaMYB221	gene20888	XP_004307600.1
FaMYB222	gene20912	XP_004307612.1
FaMYB224	gene21007	XP_004308948.2
FaMYB233	gene01828	XP_004309494.1
FaARF	gene30394	XM_004293453.2
FabHLH	gene16871	XM_004296937.2
FabZIP	gene15193	XM_004291796.2
FaC3H	gene01153	XM_004304028.2

FaGRAS1	gene13212	XM_011472024.1
FaGRAS2	gene29558	XM_004300565.2
FaHD-ZIP1	gene18656	XM_004306582.2
FaHD-ZIP2	gene25942	XM_004299351.2
FaMADS	gene24494	XM_004309676.2
FaNAC1	gene31149	XM_004291619.2
FaNAC2	gene31150	MN530979.1
FaTALE1	gene18718	XM_004302650.2
FaTALE2	gene20865	XM_004307537.2
FaTrihelix	gene01828	XM_004309446.2
FaWRKY1	gene01340	KU207054.1
FaWRKY2	gene23963	XM_011465135.1
FaWRKY3	gene28720	XM_004303826.2

Table S3. Primers used in RT-qPCR.

Genes	Forward primer	Reverse primer	Product length
FaRIB413	TACTGCGGGTCGGCAATCGGACG	ACCGTTGATTCGCACAATTGGTCATCG	150
FaGAPDH	GAGTCTACTGGAGTGTTCA	CTTGTATTCGTGCTCATTCA	135
FaQR	GAGTCCATACCCTCTGTAAATAAGGC	GAGAGTCAGTGTCCTTGAAGTAACC	192
FaQR3	GTCTAGAAACCTCTGCCCTTGAAA	TGATCAGCACCTGATCCTCTTTAAT	191
FaQR1/2/4	TAGAAACCTCTGCCCTGGCAAG	TGATCAGCACCTGATCCTCTTTAAC	189
FaAGO4	CAAAATAACTGGATTGAGCGAGAG	GGGGATGTATGTTGGACGC	202
FaDML1	AATCTCCAGCCACACCGTATCC	CTCTCACTGTCGCTTTTCCTTCTG	102
FaDML2	GTCCGCCACCAAGAACCCAAGA	TGAGGTGACAAATGGCTGTTTCTGA	258

Table S4. Primers for DNA methylation detection.

Region	Forward primer	Reverse primer	Product length	Position
FaQR1Pro-M1	TAGAGTTGGGTGGGTAATGGTTGAG	TTACCACTRCTTTTACCACTCACA	433	-532 ~ -100 bp
FaQR2Pro-M1	GAGTATGAATATYGATGAAGAGTGA	AATTCTRRATTTACCCTCTCTTTTT	458	-822 ~ -365 bp
FaQR3Pro-M1	TGYTTYGAGGYATGAAGGTTGAATGTAG	ATCAARTRCTCRTCACATTTA	382	-2325 ~ -1944 bp
FaQR3Pro-M2	TGTGTTGAYGATTTGTAGAAGGAAG	CACTATAAATATCCCCTCTCATATC	493	-1910 ~ -1418 bp
FaQR3Pro-M3	GTYTTAGTTGATYGATTTAATTATG	CACATTTATCTCTTRAARCTCATTA	397	-1289 ~ -893 bp
FaQR3Pro-M4	GATAGAGTATTYGGGATATTGTGAG	CCCACCTTTCRATCTTATATCTACA	382	-837 ~ -456 bp
FaQR4Pro-M1	GAGGAATTATTAAGGGAGAAGAGGG	CCCATCRAAACTCTATCTCTAAATA	436	-1894 ~ -1459 bp
FaQR4Pro-M2	TTGGTTTGATTTTTTGAATTTGTTT	TCCTAAAAAACTTACCCATATTTTCTC	437	-1097 ~ -661 bp
FaQR4Pro-M3	TTAGTAATTTAGAGAGGGGTAAAAT	AAATAAATTTTCTACTAAAAATTCACTC	154	-365 ~ -212 bp
FaQRCDS-M1	TTGAGAAYYTTGGGTGTTGATTTGG	ATRRRATACACAACCACCTTTCC	260	-703 ~ -962 bp

Table S5. Primers for vector construction.

Name	Forward primer	Reverse primer	Product length
FaQR1Pro	GCGTTACCACTGCTTTTACCACTCACAG	TGTTAGACGGTGTATTGCAAAAGTAA	535
FaQR2Pro	GGAACACCATCAGGAGCAACAGACTCTGC	TGTTAGACGGTGTATTGCAAAAGTAA	1085
FaQR3Pro	TGCTTCGAGGCATGAAGGTTGAATGTAG	TGTTAGACGGTGTATTGCAAAAGTAA	2325
FaQR4Pro	TGAGGGGGTCTGAATCCTATTCCCATCG	TGTTAGACGGTGTATTGCAAAAGTAA	1915
FaAGO4-RNAi	GGGGACAAGTTTGTACAAAAAAGCAGGCTATGGATTCATTTGAGCCAGATGGAA	GGGGACCACTTTGTACAAGAAAGCTGGGTCAGAACACTGTAATGGAAGAAGTGT	324
FaDML1-RNAi	GGGGACAAGTTTGTACAAAAAAGCAGGCTGATCCAGGAGATGACATATCCTCAT	GGGGACCACTTTGTACAAGAAAGCTGGGTTGCCTCCATGGAACTTTTTCTGGCA	261
FaDML2-RNAi	GGGGACAAGTTTGTACAAAAAAGCAGGCTATGGATATGAATGAGCAGAGGAAAG	GGGGACCACTTTGTACAAGAAAGCTGGGTATCCACATTTGAGCTTGCCTTCTCT	399
FaAGO4-SK	cgcggtggcggccgctctagaATGGATTCATTTGAGCCAGATGG	tgatttcagcgaattggtaccTCAGCAGAAAAACATAGAATTGGC	2835
FaQR3-LUC	ctatagggcgaattgggtaccTGTGTTGACGATTTGTAGAAGGAAGTT	tgtttttggcgtcttccatggTGTTAGACGGTGTATTGCAAAAGTAA	1910
FaQR3-LUC	ctatagggcgaattgggtaccTCGATGCTCCTCCGTAAATGGAATGC	tgtttttggcgtcttccatggTGTTAGACGGTGTATTGCAAAAGTAA	1418
FaMYB014-SK	cgcggtggcggccgctctagaATGGCTGACTTGGATCACTCT	tgatttcagcgaattggtaccTCATTCACTTGTTGAGTATCTTGAA	225
FaMYB016-SK	cgcggtggcggccgctctagaATGGCTATGAATCGGAAGGAAAT	tgatttcagcgaattggtaccTCACTCAATTCTGCTGATCCCA	930
FaMYB017-SK	cgcggtggcggccgctctagaATGGAGCTCAGCTTAGATTCCA	tgatttcagcgaattggtaccCTACACATCTCCACTGGAGTTGTG	1104
FaMYB019-SK	cgcggtggcggccgctctagaATGGGTGCTCCTAAGCAGAAGT	tgatttcagcgaattggtaccTTACCCGTGTGCGAGTTTCATA	951
FaMYB034-SK	cgcggtggcggccgctctagaATGGGTGCTCCTAAGCAGAAAT	tgatttcagcgaattggtaccTTACATGGACTTTACCATCTTTCCA	897
FaMYB035-SK	cgcggtggcggccgctctagaATGGGGAGATCTCCATGCTGT	tgatttcagcgaattggtaccTTATTTCATCTCCAAGCTTCTGTAATC	801

FaMYB036-SK	cgcggtggcggccgctctagaATGGTGAGAGCTCCGTGCT	tgatttcagcgaattggtaccTCATATATCTAGCAATTCTGGAATTTCC	828
FaMYB037-SK	cgcggtggcggccgctctagaATGGAGGCACGCCCTGC	tgatttcagcgaattggtaccCTATTCTTTAATTTTGGGACGCTTAGG	1542
FaMYB040-SK	cgcggtggcggccgctctagaATGGCCGCTCCACCCTCA	tgatttcagcgaattggtaccTCAATCGAGAGCGAACGCC	840
FaMYB045-SK	cgcggtggcggccgctctagaATGCCAGCTTCTCCTTCCTT	tgatttcagcgaattggtaccTCAACCTAAACCAGAGCTAGTACCT	2379
FaMYB047-SK	cgcggtggcggccgctctagaATGGGAAGAGCTCCATGTTGTG	tgatttcagcgaattggtaccTCAGCAGAAAAAGAAATCAGCATC	834
FaMYB049-SK	cgcggtggcggccgctctagaATGGCTTCTTCTTCAACCAAGA	tgatttcagcgaattggtaccCTACTCCACCTTGCTAATCCCA	981
FaMYB051-SK	cgcggtggcggccgctctagaATGGACGGTGATAAAAATAGTTATACTCC	tgatttcagcgaattggtaccTTATCTGCATCCCTTCAACAAATAG	3252
FaMYB055-SK	cgcggtggcggccgctctagaATGGGAAGGAAGGAGATGACATC	tgatttcagcgaattggtaccTCAATCAACTGCAGGTTTATTAGCA	1836
FaMYB060-SK	cgcggtggcggccgctctagaATGACTACCAAATCGATCCAACAG	tgatttcagcgaattggtaccCTAGCTTTTGGTTTTAGGTGCAGA	1422
FaMYB062-SK	cgcggtggcggccgctctagaATGGGTCGTTCTCGTGGAAA	tgatttcagcgaattggtaccTCAAGACTGTGCTATCCCCTTTT	1602
FaMYB064-SK	cgcggtggcggccgctctagaATGTTTCCGAGATTGGTGCAG	tgatttcagcgaattggtaccCTATATAGCATACCTAGTCATTCCATGAG	864
FaMYB065-SK	cgcggtggcggccgctctagaATGGACAATATCAATGGAGCCA	tgatttcagcgaattggtaccCTACATATCACTCCCAGCAACCTG	882
FaMYB068-SK	cgcggtggcggccgctctagaATGGTGAGAACTCCTTGCTGTGATG	tgatttcagcgaattggtaccTCATGTTGCTCCGTACAAGAAATCTC	801
FaMYB070-SK	cgcggtggcggccgctctagaATGGTGAGGGGTAGTACTCAG	tgatttcagcgaattggtaccTCACATTCCATTTGTTTCATCAGATTGTA	780
FaMYB074-SK	cgcggtggcggccgctctagaATGGGCGACCTAACGGAATC	tgatttcagcgaattggtaccCTATAGCATACAGAACAAAGCTGACAGA	957
FaMYB078-SK	cgcggtggcggccgctctagaATGAAGTTGGAAATGGGAATTCTCTC	tgatttcagcgaattggtaccTCATCCATGCAGGTAGTGGT	906

FaMYB088-SK	cgcggtggcggccgctctagaATGAGGAACCCATCTTCGTCTT	tgatttcagcgaattggtaccCTAATGAAGTGGCGTGAAATGATA	375
FaMYB091-SK	cgcggtggcggccgctctagaATGAATCTTCAAAACGGTCGAG	tgatttcagcgaattggtaccCTACACAGGAATATTGTCCAAGGA	2061
FaMYB093-SK	cgcggtggcggccgctctagaATGTATCACGCAAAGAAATTCTCG	tgatttcagcgaattggtaccCTATTGTTCAGAGTCACACAACGGA	939
FaMYB094-SK	cgcggtggcggccgctctagaATGGTGAGAGCTCCTTGCTG	tgatttcagcgaattggtaccTCAAAACTCTGGTAGTTCTGGTGTG	771
FaMYB098-SK	cgcggtggcggccgctctagaATGCAGGGTTCAAAGCTTGGT	tgatttcagcgaattggtaccTTAACACACAGGAGGGGAGGC	1215
FaMYB103-SK	cgcggtggcggccgctctagaATGAGGAAACCCTGCTGCGA	tgatttcagcgaattggtaccTTATCTGAAGAGAGGAAGCGTGGTA	768
FaMYB110-SK	cgcggtggcggccgctctagaATGTCTCGCACGTGCTCTCAG	tgatttcagcgaattggtaccTCAAGCCACACTGATGATGCTAT	996
FaMYB114-SK	cgcggtggcggccgctctagaATGGACTCCGACGTCGACG	tgatttcagcgaattggtaccCTATTTAGTCATGTTTCTCCACTTGTCC	2016
FaMYB115-SK	cgcggtggcggccgctctagaATGGGAAGGCAACCTTGCTG	tgatttcagcgaattggtaccTTAAATTTTCTCAATCCCTAATGTGTC	840
FaMYB122-SK	cgcggtggcggccgctctagaATGGCTGGTGTTGCAAACAGT	tgatttcagcgaattggtaccTCACATCCCCTGCATGTTCC	852
FaMYB123-SK	cgcggtggcggccgctctagaATGGAAGACAAACGCGGCG	tgatttcagcgaattggtaccTTACTTTGCCCCCATAGAAGAAA	3009
FaMYB126-SK	cgcggtggcggccgctctagaATGTCTTGCAACGCTGCTTG	tgatttcagcgaattggtaccTTATTGGTGCATTGTGGGATGTG	912
FaMYB135-SK	cgcggtggcggccgctctagaATGCCTCATAAAGGGATTTTGG	tgatttcagcgaattggtaccTTAGTCCAGTACTTCTTTGCTGTGC	1416
FaMYB137-SK	cgcggtggcggccgctctagaATGAGGAAGCCCTGCTGCG	tgatttcagcgaattggtaccTTAAGCAACTTGAGGATCAGCCATT	567
FaMYB139-SK	cgcggtggcggccgctctagaATGTCATCCTCCGGCACCTG	tgatttcagcgaattggtaccTCAAGCAACACTGATGATGTTGTTG	894
FaMYB141-SK	cgcggtggcggccgctctagaATGATGATAGATGATAACAACAACGCT	tgatttcagcgaattggtaccTTATACCACCTTTCTCTCATCACCTG	1665
FaMYB142-SK	cgcggtggcggccgctctagaATGGGGAGGTCACCTTGCTG	tgatttcagcgaattggtaccCTATGAATTCAAGGGTCTGCGAA	942
FaMYB146-SK	cgcggtggcggccgctctagaATGGATGTGAAGGATCTCACCTTG	tgatttcagcgaattggtaccCTAATTAACTTGGCCTCTCCCCA	468
FaMYB147-SK	cgcggtggcggccgctctagaATGGTTTGCACTGCCAGTGAT	tgatttcagcgaattggtaccTCAACGGGAGTTAGAGCCGTC	1746
FaMYB148-SK	cgcggtggcggccgctctagaATGTCCTCCCCCACCGCC	tgatttcagcgaattggtaccCTAGGAATCAGTTGGCACAGCCTT	792
FaMYB159-SK	cgcggtggcggccgctctagaATGGAGGGTTATTTCGGTGTGA	tgatttcagcgaattggtaccTCATACGTAGGAGATGTTGACTAGA	708

		TCA	
FaMYB160-SK	cgcggtggcggccgctctagaATGGGGAGGCATTCTTGTTGCTACAAG	tgatttcagcgaattggtaccTTAAAGGGTCTGTCCAAAAGCTACT	1326
FaMYB162-SK	cgcggtggcggccgctctagaATGGCGCCCACCAGAAAA	tgatttcagcgaattggtaccTCAAGTTGGCACGAGTGCTAAT	3648
FaMYB164-SK	cgcggtggcggccgctctagaATGGACGGTCATTATCCTCACC	tgatttcagcgaattggtaccTTACATGCCCCCTCTATTAAGCC	948
FaMYB166-SK	cgcggtggcggccgctctagaATGTATAGGGGAATTGAAGTTCTATCTC	tgatttcagcgaattggtaccTCACTGATATTGCATCTCGAAACG	942
FaMYB167-SK	cgcggtggcggccgctctagaATGGAACCGGAACTGGCC	tgatttcagcgaattggtaccTTAGGTTGATAGCACATTAGTCTTCATG	1161
FaMYB170-SK	cgcggtggcggccgctctagaATGTCGTCCTCCGACGGG	tgatttcagcgaattggtaccCTAATAATCTCCTGGTTGTGGCTG	831
FaMYB171-SK	cgcggtggcggccgctctagaATGACTCGGCGCTGCTCCC	tgatttcagcgaattggtaccTCAGACTGCTTGGATGACACTGGTC	1077
FaMYB172-SK	cgcggtggcggccgctctagaATGGATGACACGGAAGATGATG	tgatttcagcgaattggtaccTTACCCACTAGCATTTTCAGCAGA	1188
FaMYB173-SK	cgcggtggcggccgctctagaATGGAAATGGAGGACCAGTACAA	tgatttcagcgaattggtaccTTAACTACCCTTGCTGAGCTTCAA	1740
FaMYB174-SK	cgcggtggcggccgctctagaATGAGGGAAGACGACTCCAATT	tgatttcagcgaattggtaccTCAATTATCCCCAGTAGGAAAAAGA	963
FaMYB187-SK	cgcggtggcggccgctctagaATGGAAGCAGTGTTTGTGGTTGA	tgatttcagcgaattggtaccTTACAAGTAGCCTGATCTTGTTTGATG	1824
FaMYB189-SK	cgcggtggcggccgctctagaATGGCTCCAAAGAGAGAAGAGTCC	tgatttcagcgaattggtaccCTAGTAAAGGAACTTGTTGACCCATT	687
FaMYB195-SK	cgcggtggcggccgctctagaATGGATTGCGAATCGGTTCA	tgatttcagcgaattggtaccTTAGGAGAGCTTGGGTACCCC	813
FaMYB196-SK	cgcggtggcggccgctctagaATGAAGGAGCGGCAGCGC	tgatttcagcgaattggtaccCTAGTATGAGAAGGATGGCCATGA	1020
FaMYB199-SK	cgcggtggcggccgctctagaATGGGCAGACCTCCTTGCTGT	tgatttcagcgaattggtaccTCAGAAGAAGCCGGCATTTTCA	1083
FaMYB200-SK	cgcggtggcggccgctctagaATGGATAAGCCCAGTTTTCCG	tgatttcagcgaattggtaccCTAGTTTCTACGCCGTTTAGCTGG	2097
FaMYB202-SK	cgcggtggcggccgctctagaATGGGCTCTGAGGCACACC	tgatttcagcgaattggtaccTCAAATTCCATTGCCAATTTTAAA	2319
FaMYB206-SK	cgcggtggcggccgctctagaATGGAGTTCAGAGATGAGAGCGG	tgatttcagcgaattggtaccTTAATGGCGCTTTGCATTATTGA	1656

T

FaMYB209-SK	cgcggtggcggccgctctagaATGGAACAATATCAACAACACGGA	tgatttcagcgaattggtaccTTACCTTCTTGATCCCTTTTTGGG	2016
FaMYB214-SK	cgcggtggcggccgctctagaATGGTGTCTGTAAACCCAAACCC	tgatttcagcgaattggtaccCTAAGTAGACAGGATGGCATTCTCTG	1026
FaMYB218-SK	cgcggtggcggccgctctagaATGTTCCAGGAAGCGCCGCCGTGTT	tgatttcagcgaattggtaccTCACTTTGGGAAACCAAAATCTTGA	672
FaMYB219-SK	cgcggtggcggccgctctagaATGGAGAAAGAGGGTAACCAGGAT	tgatttcagcgaattggtaccTTAACTTCTTCCAATTCTTAATGAAGAA	1005
FaMYB221-SK	cgcggtggcggccgctctagaATGACACTCCCAAACGACG	tgatttcagcgaattggtaccCTAGCTCAATCCCTTAACTTCAGTTG	1170
FaMYB222-SK	cgcggtggcggccgctctagaATGGCTGATACTAATTGGTTTTTGC	tgatttcagcgaattggtaccTCATCCATGTATCTGATGTCTTGAGG	888
FaMYB224-SK	cgcggtggcggccgctctagaATGGAGCGCCCGACCCGG	tgatttcagcgaattggtaccCTACTCATGTACACACAACATAGGCC	855
FaMYB233-SK	cgcggtggcggccgctctagaATGCAAGGAGGTGGAGGAGG	tgatttcagcgaattggtaccTCACTGAACCATTGCCAAGAAGG	2310
FaARF-SK	cgcggtggcggccgctctagaATGAGGCTCTCTTCTGCTGGC	tgatttcagcgaattggtaccTCAATACTCGAGTGATCCCACCG	2643
FabHLH-SK	cgcggtggcggccgctctagaATGGAAAAGGACAACAGTTCAGG	tgatttcagcgaattggtaccTTAATGCTCAACTTTCATCTGAGCTG	1653
FabZIP-SK	cgcggtggcggccgctctagaATGGCTACTTCGAGCGGAAA	tgatttcagcgaattggtaccTTAGTACTGCAGCATGTCTGCTGTG	516
FaC3H-SK	cgcggtggcggccgctctagaATGCCAGACACTCGGCAGG	tgatttcagcgaattggtaccTCAACCAGATTGGTCTTGTACAGTTT	1314
FaGRAS1-SK	cgcggtggcggccgctctagaATGCAATCAGGGTTCAATTCCG	tgatttcagcgaattggtaccTTAACGCCAAGCTGATGCCA	2031
FaGRAS2-SK	cgcggtggcggccgctctagaATGAGAGTACCCGTCACCACACC	tgatttcagcgaattggtaccCTAACACCTCCATGCCGACG	1659
FaHD-ZIP1-SK	cgcggtggcggccgctctagaATGTTTGAGTCAGACCAAGTTGAATAT	tgatttcagcgaattggtaccTCAAGACCAAAAATTCCACCACT	732
FaHD-ZIP2-SK	cgcggtggcggccgctctagaATGGCCGGCGGTGGGAGG	tgatttcagcgaattggtaccCTAGTAAGACCAGGACCAAAAGGC	981

FaMADS-SK	cgcggtggcggccgctctagaATGGAGATCCCAAAACAAATCACA	tgatttcagcgaattggtaccTCAAACAAGTTGGAGAGGTGTCAG	750
FaNAC1-SK	cgcggtggcggccgctctagaATGGGCGTTCCGGAAACC	tgatttcagcgaattggtaccTCACTTTCCACTGCCAAAGCT	1065
FaNAC2-SK	cgcggtggcggccgctctagaATGGAGAGCACCGACTCGTCT	tgatttcagcgaattggtaccCTAAGAATACCAATTCCCCGGA	1035
FaTALE1-SK	cgcggtggcggccgctctagaATGGCTGAGGGTTTTGAGCC	tgatttcagcgaattggtaccTCATCCCACAAAATCACGCA	1797
FaTALE2-SK	cgcggtggcggccgctctagaATGGCAGGCGAGGGTTTT	tgatttcagcgaattggtaccTCATTTTTTGGCCTGTAGGCTT	1221
FaTrihelix-SK	cgcggtggcggccgctctagaATGCAAGGAGGTGGAGGAGG	tgatttcagcgaattggtaccTCACTGAACCATTGCCAAGAAG	2310
FaWRKY1-SK	cgcggtggcggccgctctagaATGTTCTTCCCCGGATCATCA	tgatttcagcgaattggtaccCTAACCTAGCTGGCCTGTGTGAT	627
FaWRKY2-SK	cgcggtggcggccgctctagaATGGCCAAAGGAGGTGGACT	tgatttcagcgaattggtaccTTACTGCTGCAATTTGCTGCTG	1803
FaWRKY3-SK	cgcggtggcggccgctctagaATGTCAAATGAAAAGAAAAGCCCT	tgatttcagcgaattggtaccTCATGGCTCCTCCAGCTTGT	1107
